# Diversity, evolution and expression profiles of histone acetyltransferases and deacetylases in oomycetes

**DOI:** 10.1186/s12864-016-3285-y

**Published:** 2016-11-16

**Authors:** Xiao-Wen Wang, Li-Yun Guo, Miao Han, Kun Shan

**Affiliations:** Department of Plant Pathology and the Ministry of Agriculture Key Laboratory for Plant Pathology, China Agricultural University, Beijing, 100193 China

**Keywords:** Oomycetes, Histone acetyltransferase, Histone deacetylase, Epigenetics, Living habitats, Expression profiles

## Abstract

**Background:**

Oomycetes are a group of fungus-like eukaryotes with diverse microorganisms living in marine, freshwater and terrestrial environments. Many of them are important pathogens of plants and animals, causing severe economic losses. Based on previous study, gene expression in eukaryotic cells is regulated by epigenetic mechanisms such as DNA methylation and histone modification. However, little is known about epigenetic mechanisms of oomycetes.

**Results:**

In this study, we investigated the candidate genes in regulating histone acetylation in oomycetes genomes through bioinformatics approaches and identified a group of diverse histone acetyltransferases (HATs) and histone deacetylases (HDACs), along with three putative novel HATs. Phylogenetic analyses suggested that most of these oomycetes HATs and HDACs derived from distinct evolutionary ancestors. Phylogenetic based analysis revealed the complex and distinct patterns of duplications and losses of HATs and HDACs in oomycetes. Moreover, gene expression analysis unveiled the specific expression patterns of the 33 *HATs* and 11 *HDACs* of *Phytophthora infestans* during the stages of development, infection and stress response.

**Conclusions:**

In this study, we reveal the structure, diversity and the phylogeny of *HATs* and *HDACs* of oomycetes. By analyzing the expression data, we provide an overview of the specific biological stages of these genes involved. Our datasets provide useful inputs to help explore the epigenetic mechanisms and the relationship between genomes and phenotypes of oomycetes.

**Electronic supplementary material:**

The online version of this article (doi:10.1186/s12864-016-3285-y) contains supplementary material, which is available to authorized users.

## Background

In eukaryotes, gene expression and physiological function can be regulated by epigenetic mechanisms. Epigenetic modifications of chromatin can cause heritable changes that are not encoded by the underlying DNA sequences. The mechanisms involved mainly include DNA methylation and histone modification [1].

DNA methylation mostly occurs at CpG dinucleotides through adding methyl groups and converting cytosine to 5-methylcytosine by DNA methyltransferases (DNMTs) [2, 3]. DNMT1, DNMT2, DNMT3A, DNMT3B, and DNMT3L are C(5)-cytosine-specific DNA methyltransferases [4–6]. A research has shown that promoters with more 5-methylcytosines have lower transcriptional activity [7]. However, no evidence of the regulation of gene expression associated with DNA methylation in oomycetes is documented [8, 9].

Histone proteins are the core particles of nucleosomes that are packed together to form eukaryotic chromatins [10]. The N-terminal tails of histones can extend from the nucleosomes and can be modified post-translationally (PTMs) by mechanisms such as acetylation, methylation, phosphorylation, ubiquitination, and sumoylation by different histone modifiers [11]. Among the PTMs, histone acetylation at lysine residues is well characterized in yeast, plants and animals [12, 13]. Histone acetylation occurs at the amino groups of the lysine residues on the N-terminus of histone tails. The dynamic PTM histone lysine acetylation is regulated by histone acetyltransferases (HATs) and histone deacetylases (HDACs) [13–15]. HATs transfer an acetyl moiety from acetyl coenzyme A (acetyl CoA) to the Ɛ-amino group of specific lysine residues on histone N-terminal tails [16, 17]. As a consequence, histone acetylation promotes RNA polymerase and other transcriptional factor complexes to interact with DNA [18], and leads to increased gene expression [19]. In contrast, HDACs remove acetyl modifications from histones and lead to down-regulated gene expression. Gene families of both HATs and HDACs are evolutionarily conserved in eukaryotes [20–22] and have been shown to play a crucial role in transcriptional regulation in yeast, plants and mammals [11].

Based on intracellular localization and substrate specificity, HATs are classified into two categories, type A and type B [23]. The type A HATs are usually localized in the nuclei and are involved in transcriptional regulation by modification of specific lysine residues on histone tails [24]. According to sequence conservation in the functional domains of histone acetyltransferases and their functions, type A HATs can be further classified into different families, such as GNAT, Elp3, Hpa2, MYST, p300/CBP, TAF_II_250 and ACTR/SRC-1 [25]. Type B HATs are located in the cytoplasm, where they can acetylate free histones prior to assembly into nucleosomes, such as Hat1 in yeast [26]. Each family of HATs has its own highly conserved sequence [27].

HDACs are divided into four phylogenetic groups, Class I, II, III and IV, according to localization and tissue-specific expression [28]. In addition, HDACs can be classified into two types based on their dependence on zinc or NAD co-factors [29, 30]. The Class I, II and IV HDACs are zinc-dependent, while the Class III HDACs are NAD-dependent.

Previous studies have found that the core domain of HATs plays an especially important role in histone substrate catalysis, while the N- and C-terminal domains are key elements in histone substrate binding [31]. Analysis of acetylated lysines on histone revealed that the region of histone acetyltransferase domains interacting with peptide substrates typically do not exceed 14 to 20 amino acid (aa) in length flanking each lysine [31] (7 to 10 residues on each side). In addition, an analysis of frequency distribution of amino acids surrounding acetylated and non-acetylated lysines in histone indicated that residues surrounding acetylated lysine were usually small and polar, while basic, acidic and hydrophobic residues were more inclined to surround non-acetylated lysine [31]. Based on these findings, Basu et al. [32] developed the program PredMod for predicting the acetylated sites.

The Oomycetes are a group of fungus-like eukaryotes with diverse microorganisms living in marine, freshwater, and terrestrial environments [33, 34]. Many members of oomycetes are important pathogens of plants and animals, causing severe economic losses. Examples include *Phytophthora infestans*, the causal pathogen of potato late blight, which resulted in the death of millions of people in the Irish potato famine in the 19^th^ century; the sudden oak death pathogen *Phytophthora ramorum* and Jarrah forest dieback pathogen *Phytophthora cinnamomi*, which affect a large variety of woody plants resulting in natural and ecosystems damage [35, 36]; and *Saprolegnia parasitica,* a devastating pathogen of many freshwater fish [37]. Although most oomycetes have nutritional and ecological characteristics similar to the true fungi, several biochemical and cytological features distinguish them from the true fungi [38]. For instance, (i) their cell walls are composed of cellulose and glycan instead of chitin; (ii) their mitochondria contain tubular cristae instead of disc-like cristae; (iii) their nuclei are diploid in asexual stage; and (iv) they are sterol auxotrophs. Concrete evidence from molecular phylogeny has firmly established their distinct taxonomic position as a specific group of eukaryotes belonging to the phylogenetic lineage of biflagellate “heterokont” organisms universally referred to as “Stramenopila”, with photosynthetic algae such as brown algae and diatoms [39]. Stramenopiles and Alveolates, which include the apicocomplexa, ciliates and dinoflagellates, compose the superkingdom Chromalveolates [40–43]. However, there are barely any data available on the genes and the role of epigenetic modifications in oomycetes, or even in the Stramenopiles.

Considering the importance of histone acetylation in epigenetic modifications and the existence of diverse histone acetyltransferases and deacetylases in many eukaryote species investigated, we postulated that species in oomycetes have diverse histone acetyltransferases and deacetylases. With the available genome sequences of several oomycetes species, we investigated the candidate genes of histone acetylation in ten sequenced species and provide a comprehensive overview of the structure, diversity, phylogeny and the expression pattern of *HATs* and *HDACs* of oomycetes in this study.

## Methods

### Oomycetes for database searches

**Fig. 1:**
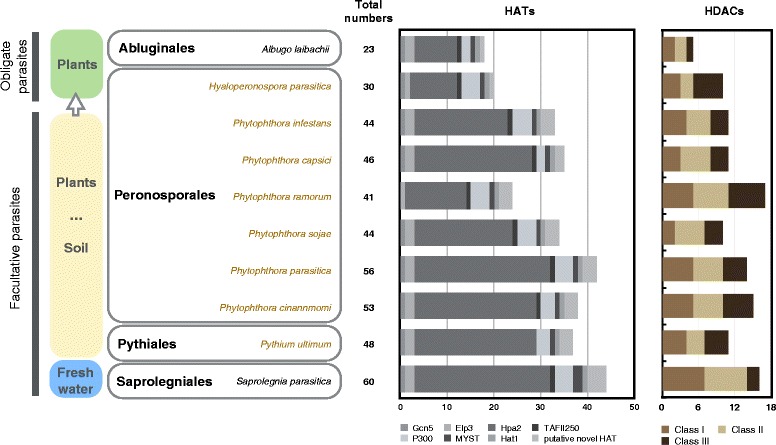
Numbers of HATs and HDACs found in ten species of oomycetes. Columns in different colors represent different families of HATs and HDACs. Species in brown are soil-borne plant pathogens

Genomes of ten species of oomycetes with divergent life styles and belonging to various taxa in oomycetes were used. They included the pathogen of fresh water fish, *S. parasitica* in Saprolegniaceae of Saprolegniales; the soil-borne plant pathogen *Pythium ultimum* in Pythiaceae of Pythiales; the soil-borne plant pathogens *Phytophthora capsici*, *P. cinnamomi*, *P. infestans*, *P. parasitica*, *P. ramorum*, and *P. sojae*, and the obligate plant parasite *Hyaloperonospora parasitica* in Peronosporales; and the air-borne obligate plant parasite *Albugo laibachii* in Albuginaceae of Albuginales (Fig. 1). Other than *H. parasitica* and *A. laibachii*, the rest were facultative parasites.

### Gene identification and analysis of HATs and HDACs

Gene sequences and proteomes of oomycetes species used in this study were retrieved from the database of the BROAD INSTITUTE (https://www.ncbi.nlm.nih.gov/bioproject), the DOE Joint Genome Institute (http://www.jgi.doe.gov/), the EnsemblGenomes (http://www.ensemblgenomes.org/) and the *Pythium* Genome Database (http://pythium.plantbiology.msu.edu/index.html) [44–50]. Additional searches for genes of diatoms (*Phaeodactylum tricornutum*, *Thalassiosira pseudonana*), ciliates (*Tetrahymena thermophila*), diplomonads (*Giardia lamblia*), yeast (*Saccharomyces cerevisiae*), human (*Homo sapiens*), Arabidopsis (*Arabidopsis thaliana*) and other species were retrieved from the NCBI, UniProt, and KEGG databases (Additional file 1). All the sequences were checked for annotation mistakes using the following methods. The structural domains of protein sequences were predicted using the SMART (http://smart.embl-heidelberg.de/) and Pfam (http://pfam.sanger.ac.uk/) databases and NCBI CD (Conserved Domains) - Search (http://www.ncbi.nlm.nih.gov/Structure/cdd/wrpsb.cgi). Additional domains were predicted and verified with the InterProScan (http://www.ebi.ac.uk/Tools/pfa/iprscan/) and ExPASy Prosite (http://prosite.expasy.org/) [51–54]. The combinations of domains in the HATs were BLAST against the databases KEGG and UniProt with default parameters (*e* value <1e-10) to search for their homologs in the genomes of other species. Signal peptides were predicted using the CBS Prediction Servers (http://www.cbs.dtu.dk/services/). The secondary structures of proteins were predicted using the online program Psipred (http://bioinf.cs.ucl.ac.uk/psipred/) [55] and CFSSP (http://www.biogem.org/tool/chou-fasman/) [56]. The sequences accession numbers and features are listed in Additional file 1. Sequence logos were created with WebLogo (http://weblogo.berkeley.edu/logo.cgi) for displaying the conserved peptides of motif A in the HATs of oomycetes [57].

### Sequence alignments and phylogenetic analysis

To infer the phylogenic history of oomycetes genes, we compared the oomycetes genes with their orthologs in diatoms (*Ph. tricornutum*, *T. pseudonana*), diplomonads (*G. lamblia*), ciliates (*Te. thermophile*), yeast (*S. cerevisiae*), human (*H. sapiens*), Arabidopsis (*A. thaliana*), and some other related species retrieved by BLAST against the genome databases with HATs and HDACs in oomycetes (*e* value <1e-10) (Additional file 1). The amino acid sequences of conserved core domains were pairwise and multiple aligned using ClustalW2 (http://www.ebi.ac.uk/Tools/msa/clustalw2/) [58]. In Pairwise Alignment, BLOSUM62 with a gap extension of 0.1 and 0.2 was used as the protein weight matrix and in Multiple Alignment, respectively. The resulted sequence alignments were used to construct phylogenetic trees with the maximum likelihood evolution algorithm in MEGA 5.22 [59]. A Poisson correction was used for multiple substitution models and pairwise deletion was used for gap split data treatment. The statistical strengths were assessed by bootstraps with 1000 replicates or replications. To investigate the events of gene duplication and loss happened during evolution of oomycetes, we constructed a phylogenetic tree of the ten species of oomycetes in this study with two diatom species (*Ph. tricornutum* and *T. pseudonana*) as out groups using the Composition Vector Tree (CVTree) based on proteomes [60]. The statistical strengths of the topology were assessed by bootstraps with 100 replicates. Then, we reconcile the HATs and HDACs ML trees with the species phylogeny using NOTUNG (v2.6; 1.5 duplication and 1 loss cost) [61, 62]. The weakly supported branches (<80% bootstrap values) were rearranged to minimize duplication/loss costs (D/L scores), and orthologous groups were formed based on duplications at the least common ancestor (Additional file 2).

### Prediction of histone acetylation sites

For each lysine of histone, probabilities of acetylation were predicted with the PredMod (http://ds9.rockefeller.edu/basu/predmod.html) [63] with the setup value of 12 as the maximum number of residues flanking each lysine. Sequence logos were created with WebLogo (http://weblogo.berkeley.edu/logo.cgi) for displaying the flanking residue distribution of the predicted acetylated and non-acetylated lysine on histone N-terminal tails [57].

### Preparation of biological material and RNA extraction

*P. infestans*, MX5-1 (A1) and YZ-6 (A1) from potato in China and 80787-94L (A2) from USA [64] were used in this study. The biological materials were obtained at various stages of asexual development by growing the isolate MX5-1 on 150 tomato rye agar plates (90-mm) and were kept at 18 °C in darkness for 10 days [64]. Sporangia (SP) sample was harvested by washing mycelia mats with sterile water, accumulated all from 30 plates then, was centrifuged for 8 min at 2,000 rpm. The sporangia suspension of other 120 plates was placed at 4 °C for 2 to 3 h to induce zoospores (ZO) formation and it was divided into four samples each consists of 30 plates as collected by different ways. The 1^st^ ZO sample was collected by centrifuge the suspension at 2,000 rpm for 8 min. The 2^nd^ sample of zoospores were induced to form cysts (CY) by vortex at 40 s and used as CY sample. Whereas the 3^rd^ Zoospores sample was incubated in pea broth at 120 rpm, at 18 °C for 3 h and the geminated cysts (GC) were collected by centrifuge at 2,000 rpm for 8 min as GC sample. The last zoospore sample was grown on pea broth at 120 rpm, at 18 °C in darkness for 48 h, and used as MY samples.

To obtain biological material at different stages of sexual development, a polycarbonate membrane with 0.4 μm pores (Millipore, Ireland, diameter 43-mm) was placed on the surface of a rye tomato agar plate (60 mm), then, isolates YZ-6 (A1) and 80787-94L (A2) were incubated 2 cm apart from each other on the polycarbonate membrane in each plate. These plates were then incubated at 18 °C in darkness. After 4, 10 and 14 days, mycelia or mycelia with oospores were harvested from the junction between the two isolates. Meanwhile mycelia were collected similarly from the single culture of A1 and A2 isolates, respectively and used as controls.

After grinding the samples with liquid nitrogen the total RNA was extracted using NucleoSpin RNA Plant kit (MACHEREY-NAGEL, Düren, Germany). The quantity and quality of RNA were measured with a Thermo Scientific NanoDrop 2000 spectrophotometer (Thermo Fisher Scientific Inc., Wilmington, USA).

### Expression analysis of *HATs* and *HDACs* in *Phytophthora infestans*

In order to gain insight into the possible role of predicted oomycetes *HATs* and *HDACs*, we examined the expression levels of 33 *HATs* and 11 *HDACs* genes of *P. infestans* through SYBR green real-time qPCR assay with specific primers designed in this study (Additional file 3). For cDNA synthesis, 1 μg of total RNA was reverse transcript with oligo(dT)_18_ primer using the Reverse Transcriptase M-MLV (TaKaRa Bio Inc. Shiga, Japan) following the manufacturer’s instructions. Before performing the real-time qPCR, the specific primers were used to amplify the target genes. The products yield were then sequenced and aligned with the target genes to ensure that the products were from the target genes. The amplified sequences that longer than 200 bp were submitted to NCBI GenBank data library under accession numbers KX492573 to KX492582 (Additional file 4). Amplifications were conducted in 25 μl volume containing 50 ng cDNA, 0.4 μM each primer, 0.2 mM dNTP, 1U rTaq DNA polymerase (TaKaRa Bio Inc. Shiga, Japan), and 17.3 μl of sterilized distilled H_2_O with the reaction conditions as follows: 95 °C for 4 min, followed with 30 cycles of 95 °C for 30 s, 60 °C for 30 s, and 72 °C for 30 s, at last 72 °C for 4 min. SYBR green real-time qPCR were performed in an ABI 7500 detection system (Applied Biosystems, Foster City, CA, U.S.A.). Amplifications were conducted in 20 μl volume containing 10 ng cDNA, 0.2 μM each primer (Additional file 3), 1× ROX Reference Dye II, 1× SYBR Premix Dimer Eraser (TaKaRa Bio Inc. Shiga, Japan), and 4.8 μl of sterilized distilled H_2_O with the reaction conditions as follows: for calculate Ct values, 95 °C for 30 s, followed with 40 cycles of 95 °C for 5 s, 60 °C for 34 s; and for obtain melt curves, 95 °C for 15 s, 60 °C for 1 min, and 95 °C for 15 s. The gene of elongation factor 1 (*ef1*, PITG_06722.1) in *P. infestans* was used as an inner control. The whole experiment was repeated with two different sets of biological samples. The 7500 system sequence detection software was used for data analysis, and the heatmaps and the HCL trees were constructed using MultiExperiment Viewer (v. 5.0). As same color can mean different expression values on different genes, comparison of colors only justified between expression levels of different development stages of the same gene.

We also analyzed the EST, cDNA, TPM and the expression profiles data of *P. infestans* from previous studies (Additional file 5). These data were retrieved from the NCBI UniGene website, and Gene Expression Omnibus DataSets [65, 66]. The expression values were log2-transformed and used to construct the heatmap.

## Results and discussion

### Identification of HATs and HDACs in oomycetes

Through a domain-based search and the analysis of the structure and core domain in comparison with the HATs and HDACs in reference species, we identified many diverse HATs and HDACs in each species, including some putative novel HATs. There are 18 and 20 HATs in the two obligate plant parasites, *A. laibachii* and *H. parasitica*, respectively, and from 24 to 44 HATs in 8 facultative parasites (Fig. 1). There are 5 HDACs in *A. laibachii* and 10 in *H. parasitica,* and from 10 to 17 in eight facultative parasites. In general, more HATs and HDACs were found in the facultative parasitic oomycetes than in the obligate parasitic ones.

### Histone acetyltransferases in oomycetes

**Fig. 2:**
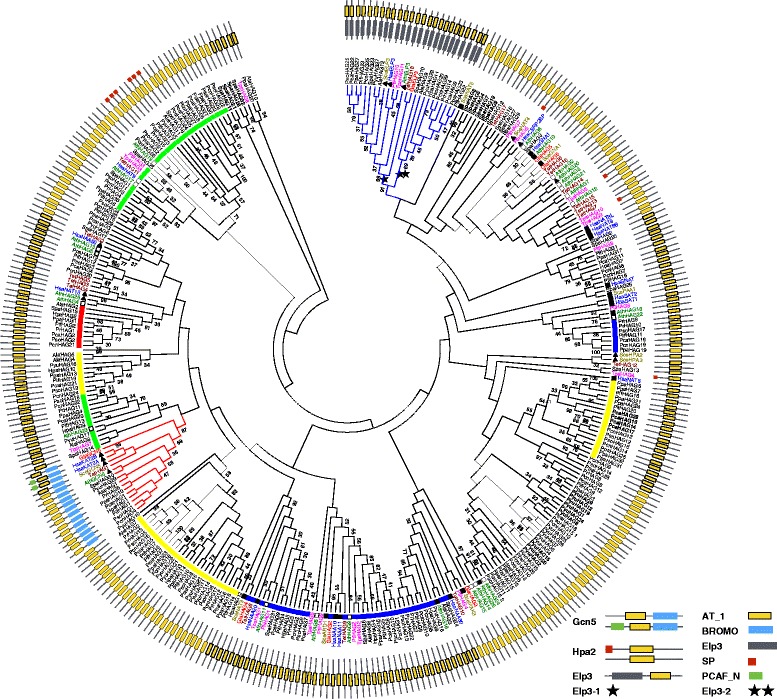
Phylogenetic tree of HAGs in oomycetes. The maximum-likelihood tree was constructed with sequences of conserved domain AT_1 of HAGs. Bootstrap values (≥30%) are shown near the tree nodes. The predicted HAGs were attributed to three known families, Gcn5, Hpa2 and Elp3. The conserved domains of each family were highlighted by different colors. In oomycetes, the Gcn5 family protein (the branch colored red) is composed of AT_1 and Bromodomain. The Elp3 family (the branch colored blue) encodes an Elp3 domain at the N-terminal in addition to the AT_1 domain, while most Hpa2s have only the AT_1 domain. Reference species are highlighted in different colors. *Pif*, *Phytophthora infestans*; *Pr*, *P. ramorum*; *Pso*, *P. sojae*; *Pca*, *P. capsici*; *Ppa*, *P. parasitica*; *Pcn*, *P. cinnamomi*; *Pyu*, *Pythium ultimum*; *Al*, *Albugo laibachii*; *Hpa*, *Hyaloperonospora parasitica*; *Spa*, *Saprolegnia parasitica*; *Hsa*, *Homo sapiens*; *Sce*, *Saccharomyces cerevisiae*; *Ath*, *Arabidopsis thaliana*; *Pti*, *Phaeodactylum tricornutum*; *Tps*, *Thalassiosira pseudonana*; *Tet*, *Tetrahymena thermophile*; *Gla*, *Giardia lamblia*

Both Type A and Type B HATs were found in oomycetes in five families, comprising HAGs, HAFs, HACs, HAMs, and Hat1s (Fig. 1). Unexpectedly, we also discovered three putative novel families of HATs with new combinations of conserved domains. The HAG group consists of Gcn5, Elp3 and Hpa2. Gcn5 is recognized in most oomycetes species, except *P. ramorum* (Fig. 1). It contains two conserved domains: a histone acetyltransferase domain (Acetyltransf_1 (AT_1) PF00583) of 90 aa at the N-terminus, and a bromodomain of 110 aa (PF00439) at the C-terminus [67] (Fig. 2). Sequence alignment reveals that oomycetes Gcn5 is highly conserved with 72.4% identity. Two proteins from the Elp3 family, Elp3-1 and Elp3-2, were recognized in most oomycete species, although only one was found in *P. ramorum* and *H. parasitica*. These proteins have an AT_1 domain at the C-terminus and a 260-aa Elp3 domain (SM000729) at the N-terminus. Unlike the Gcn5 and the Elp3 families, Hpa2 family has multiple members, ranging from 9 in *A. laibachii* to 29 in *P. parasitica* and *S. parasitica* (Fig. 1). Many Hpa2 proteins have only the AT_1 domain, but a few also have a signal peptide at their N-terminal region, similar to some Hpa2s in diatoms (Fig. 2). Only one protein of the HAFs family, TAF_II_250, was recognized in most oomycetes species, except in *Py. ultimum*. The approximately 500 aa long HAF consists of an AA_kinase (PF00696) domain at the N-terminus and an AT_1 domain at the C-terminus (Additional file 6). Two to four HACs that are orthologs of the p300/CBP histone acetyltransferase family are encoded in each oomycete genome. Their domain compositions are variable in the presence of the ZnF-TAZ (SM000551) domain. Most of them contain a KAT11 (SM001250), a BROMO (PF00439) and a PHD (SM000249) domain (Additional file 7). KAT11 is an ortholog of the fungal KAT11 protein (previously known as RTT109), which is required for H3K56 acetylation [68]. Besides, most oomycetes encode one MYST acetyltransferase in the HAM family, except for *S. parasitica,* which has two. HAM is approximately 460 aa long and is recognized by the N-terminal Chromo (PF00385) domain and the C-terminal 200 aa MOZ-SAS (PF01853) domain (Additional file 8). Moreover, all of the oomycetes species analyzed here has a homologous protein of the Hat1_N family, namely Hat1. The Hat1 protein contains a conserved Hat1_N (PF10394) domain, which is the same as the N-terminal part of the AT_1 domain of histone acetyltransferase. As an exception, the *S. parasitica* Hat1 (XM_012343809.1) carries an additional AT_1 domain similar to AthHat1 (NM_125057.3) in Arabidopsis (Additional file 9).

During our investigation, three novel families of HATs were revealed in oomycetes through combinations of domains. All three have a common functional AT_1 domain; as illustrated, PifHAT1 and its homologs (I) also contain an extra N-terminal LCM (PF04072) domain. Similarly, PifHAT2 and its homologs (II) have a Fascin (cl00187) and a DUF706 (PF05153) C-terminal domain, while PifHAT3 and its homologs (III) have an additional C-terminal PhzC-PhzF (PF02567) domain (Additional file 10). Such joint synchronization has not been reported previously and we did not detect additional homologs other than one PifHAT3 (XM_002895006.1) in the genome of *T. pseudonana*, a diatom. In addition, 2–3 putative novel HATs were found in most facultative parasites; while only one was identified in each of the two obligate parasites *H. parasitica* and *A. laibachii*.

**Fig. 3:**
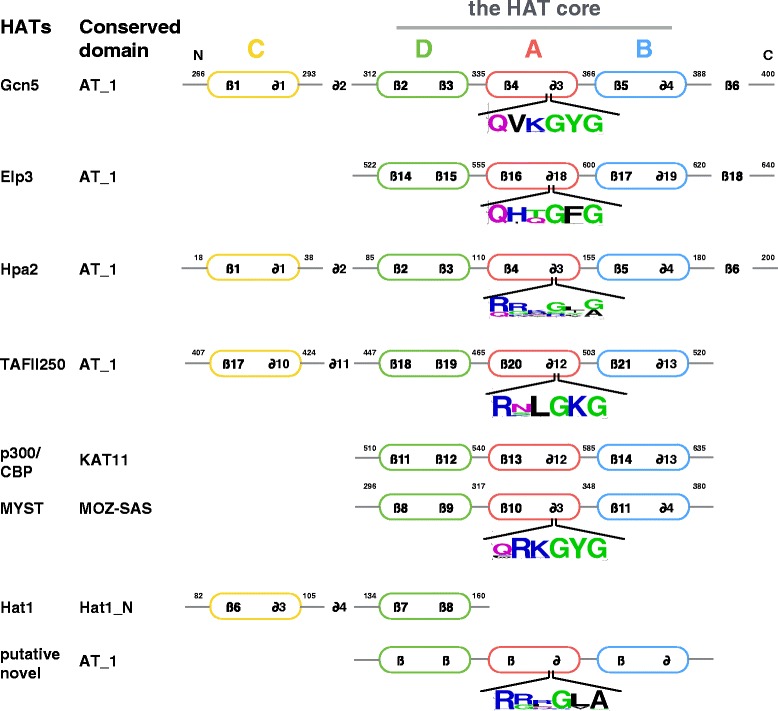
Conserved motifs identified in different families of HATs in oomycetes. Motif A of most of the HATs identified has a highly conserved peptide Arg/Gln-X-X-Gly-X-Gly/Ala. The overall height of the stack predicted by the WebLogo indicates the sequence conservation at each amino acid, while the height of symbols within the stack indicates the relative frequency of each amino acid at that position

Secondary structural predictions suggest that most of HATs have conserved motifs in its conserved domain. Except Hat1, oomycetes HATs have a conserved core region, including motif D, A and B, for the function of histone acetyltransferase (Fig. 3). In addition, a motif C is present at the N-terminus of protein Gcn5s, Hpa2s, HAFs, and Hat1s. Furthermore, the motif A has a conserved peptide Gln/Arg-X-X-Gly-X-Gly/Ala, which associates with acetyl-CoA recognition and binding. However, like the KAT11 domain of HACs in human and Arabidopsis [27], no conserved peptide was found in motif A of KAT11 of oomycetes. Similar to the functional Hat1p (NM_001183815.1) in *Saccharomyces cerevisiae* [69], the Hat1_N domain of oomycetes Hat1s contains only motifs C and D.

### Histone deacetylases in oomycetes

**Fig. 4:**
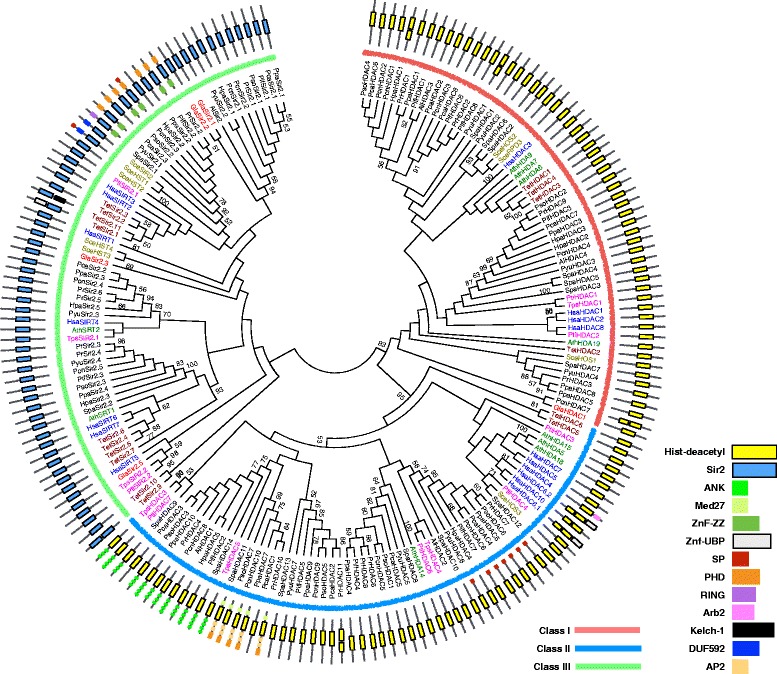
Phylogenetic tree of HDACs in oomycetes. A maximum-likelihood phylogenetic tree was constructed with sequences of HDAC conserved domains from the species described in Fig. 2. All the predicted HDACs were attributed to three known classes (I, II and III) based on the combination of domains. Conserved domains of each family and reference species were highlighted by different colors and bootstrap values (≥50%) are shown near the tree nodes

Only three Classes (I, II & III) of HDACs are identified in oomycetes (Figs. 1 and 4). For Class I HDACs, two to seven members occurred in each species. The protein structures are simple containing only the conserved core domain, the Hist_deacetyl (PF00850) of histone deacetylases (Fig. 4). For class II HDACs of oomycetes, two to seven members were found in each species. With the Hist_deacetyl domain these members have in common, some contain new conserved domains that have not been documented in the known histone deacetylases, such as a repeat of 3 to 4 ANK domains (SM000248), which could form a helix-loop-helix structure involved in protein-protein interaction (Fig. 4), or a combination of a C-terminal Med27 domain (PF11571) and a N-terminal cascade containing a PHD domain (SM000249) along with two AP2 domains (SM000380). The Med27 domain (PF11571) is related to regulation of the transcriptional activity of RNA polymerase II. The PHD domain (SM000249) is a C4HC3 zinc-finger-like motif thought to be involved in epigenetics and chromatin-mediated transcriptional regulation, and the AP2 domain (SM000380) is related to DNA binding. The presence of various domains on a protein indicated that these proteins could form complexes with other proteins. Similar situation was also found in some of the Class III HDACs, which contain two of the three domains including PHD, RING and ZnF-ZZ in addition to the common Sir2 domain (PF02146). One to six members of Class III HDACs are identified in each species of oomycetes.

### Phylogenetic and evolutionary analysis of HATs and HDACs in oomycetes

To investigate the evolution history of HATs and HDACs protein families in oomycetes, we compared them with orthologs of reference species including diatoms, ciliates, diplomonads, yeast, human, Arabidopsis and some other species in genome database. Phylogenetic analysis indicates that different groups of oomycetes HATs have different evolution history.

Gcn5s of oomycetes are clustered in one clade, together with the orthologs of reference species, including human, Arabidopsis, yeast, diatoms, diplomonads (*Giardia lamblia*) and ciliates (*Tetrahymena thermophila*) (Fig. 2) with high supporting value, indicating that Gcn5s are evolutionary highly conserved proteins of ancient lineage pre-dating speciation in oomycetes. HAFs of oomycetes are clustered in one clade that close related to the clade containing diatoms and red algae (*Galdieria sulphuraria*, *Chondrus crispus* and *Cyanidioschyzon merolae*) (Additional file 6). This result indicated that oomycetes HAFs were obtained in ancient times before speciation in oomycetes, and they had a common ancestor with that of red alga and diatoms. The lack of *P. ramorum Gcn5* and *Py. ultimum HAF* in the genome database is probably due to the deficiencies in gene assembly as *Gcn5* (PYU1_T008661) of *Py. ultimum* was also absent in the earlier version of genomic data.

Unlike oomycetes Gcn5s and HAFs families, phylogenetic analysis showed that members of Hpa2 proteins in each oomycetes species are split into several clades with othologs of different reference species (Fig. 2). Some (tagged with a red square in Fig. 2) are homologs of experimentally verified AthHAG26 (NM_111168.1, tagged with a black triangle in Fig. 2). Some (tagged with a blue square) are homologs of SceNAT3 (NM_001184228.1), AthHAG16 (NM_129369.5) and HsaNAA11 (NM_032693.2) (tagged with a black square in Fig. 2), which are acetyltransferases at the protein level. Some (tagged with a green square in Fig. 2) are homologs of the predicted acetyltransferases AthHAG19 (NM_128762.1) and AthHAG23 (NM_148472.2) (tagged with an empty black square in Fig. 2). These results suggested that the Hpa2 gene in oomycetes derived from different lineages in ancient times. Similar cases were also found in HACs, HAMs and HDACs of oomycetes (Additional files 7 and 8, and Fig. 4). HACs in oomycetes were split into four clades. Clades I and II are closely related to diatoms, but the other two Clades (III and IV) do not have any othologs in reference species, or in the current genome database (Additional file 7). For HAMs, most were grouped into a clade (oomycetes I in Additional file 8), with one ortholog of diatoms (XM_002176539.1) and some orthologs in marine algae belonging to Stramenopiles, disclosing their common ancestral origin. The other copy of HAM (XM_012345245.1) in *S. parasitica* was grouped into a different clade with HAMs of water molds and another copy of HAM in diatoms (XM_002176731.1). For class I HDACs, all of these oomycetes species have at least two members, and phylogenetic analysis showed that one is a homolog of diatoms; and the other is a homolog of yeast (Fig. 4). Likewise, most class II and III HDACs of oomycetes were homologs of diatoms, ciliates and diplomonads, respectively (Fig. 4).

Except the situations descried above, phylogenetic analysis showed that the Elp3-1 protein (one star in Fig. 2) is clustered with orthologs in diatoms, yeast, human and Arabidopsis, whereas the Elp3-2 protein (double stars in Fig. 2) is assembled in a separate clade without homologs in any reference species. As Elp3-1 and Elp3-2 proteins are group in the same super clades with high support value, the two Elp3 genes in each species are likely originated from the same ancient lineage, but a divergence happened later in ancient time, at least before the speciation of these species in oomycetes (Fig. 2).

Regarding the putative novel HATs identified in oomycetes based on domain compositions, we did not detect additional orthologs other than one PifHAT3 in the genome of *T. pseudonana*, a diatom (Additional file 10). Phylogenetic analysis revealed that these putative novel HATs derived from different lineage pre-dating the speciation of these oomycetes (Additional file 10).

Moreover, we found some members of HATs and HDACs with high sequence identity in many facultative parasitic species. For example, the SpaHDAC4 (XM_012357993.1) and SpaHDAC5 (XM_012345512.1), and the PrSir2.3 (DS566101) and PrSir2.4 (DS567307) have 100 and 100% identity, respectively (Fig. 4). Furthermore, aggregation of these members in a short region on the genome was observed. For example, PyuHAG2 (K3XAR0), PyuHAG15 (K3XAR1), and PyuHAG20 (K3XAQ0) with 70.5 to 90.6% identity (Additional file 11a) clustered in a region approximately 10 Kb on the genome of *Py. ultimum*. These cases indicating that duplication of HATs and HDACs happened after speciation in oomycetes. We also found the aggregation of members of HATs with various identities on the genome, such as a cluster of four genes (SpaHAG4 (XM_012338354.1), SpaHAG7 (XM_012338355.1), SpaHAG19 (XM_012338350.1) and SpaHAG9 (XM_012338349.1)) in a region of approximately 10 Kb in the genome of *S. parasitica* (Additional file 11b), suggesting that duplication and divergence of these genes happened after speciation in oomycetes. Similar cases were also found commonly in the facultative species such as *Py. ultimum* and *P. capsici,* but not in obligate parasitic species.

**Fig. 5:**
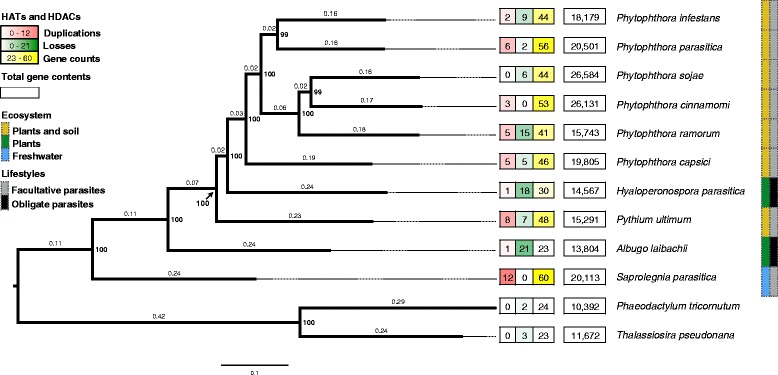
The projected events of gene duplication and loss of HATs and HDACs in ten oomycetes. Neighbor-Joining phylogeny of the analyzed oomycetes was constructed based on proteomes. The numbers of gene duplication and loss events, the total number of HATs and HDACs, as well as the predicted gene content of each species were projected in the boxes on the dash line near each branch. The robustness of the topology was assessed using 100 bootstrap replicates (*bold numbers*)

To investigate the frequency of gene duplication and loss of HATs and HDACs in oomycete evolution, we first constructed a phylogenetic tree of the ten oomycetes with closely related diatom, and then reconciled the HAGs and HDACs ML trees with the predicted species phylogeny tree. The obtained species phylogeny tree is highly supported with bootstrap values ≥99% for all nodes. It is consistent with the known evolution trends of oomycetes, clearly separating the Saprolegniales, Pythiales, Peronosporales, and Albuginales [70], as well as the six species in clades 1, 2, 7, and 8 of *Phytophthora* [71]. Both duplication and gene losses were found in *HATs* and *HDACs* of most species. In comparison with facultative plant parasitic species living in soil and plant, obligate plant parasitic species experienced less gene duplication and more gene loss events, ending up with a contraction of *HATs* and *HDACs* in their genomes (Fig. 5). The facultative parasitic oomycetes of fresh water fish, *S. parasitica* had the highest frequency (12) of gene duplication, but no gene loss (Fig. 5), gaining the most amounts of *HATs* and *HDACs* in its genome. Variation in frequency of gene duplication and loss were also observed among the 6 species of *Phytophthora*. Within the same clade, the species found more frequently in water, like *P. cinnamomi* vs. *P. sojae* had more gene duplication events, whereas, the species commonly attacking the upper plant part, like *P. infestans* vs. *P. parasitica* experienced more gene losses. Moreover, an interesting thing is that the abundance of HATs and HDACs in each species doesn’t related to the total gene content of each species, but related to the outcome of gene duplication and loss events observed in these genes, and the habitat of the species. Since the abundance of duplication events is high in those species of spending most or entire life in water, like *S. parasitica* and *Py. ultimum,* and the abundance of gene loss is high in the species of living most of or their entire life on plant, it is likely that possessing multiple copies of HATs and HDACs is favorable for life in water, but not for living on plants. Therefore, living habitat probably is an important driven force shaping the evolution of these genes.

Oomycetes have diverse fungus-like eukaryote microorganisms living in marine, freshwater, and terrestrial environments [33, 34]. Recent studies revealed that oomycetes evolved from the simple holocarpic marine parasites with two trends as, from marine water to fresh water and from marine water to soil and plant. Further, the Saprolegniales (species in fresh water), Pythiales (soil born species), Peronosporales, and Albuginales (obligate parasites on plant) (Fig. 1) are the “crown oomycetes” in these two evolution trends [70]. So, the high diversity and the evidence of gene duplication found in HATs and HDACs of the facultative parasites in Saprolegniales, Pythiales, and Peronosporales exhibits an expansion of *HATs* and *HDACs* in the evolution of oomycetes (Figs. 1 and 5), whereas the significant high frequency of gene loss outcome the gene duplication events possibly reveal the contraction of *HATs* and *HDACs* in obligated plant parasitic species in the process of adapting to plant. In general, the adequacy and diversity of HATs and HDACs found in oomycetes reflects the evolution of these organisms with the change of habitat.

Previously, several large-scale duplications in genome of several plant parasitic *Phytophthora* [66, 72] and constantly gene duplication and loss has been found in the genomes of pathogenic oomycetes [73]. The rapid expansion and diversification of many protein families related to plant infection, such as hydrolases, ABC transporters, protein toxins, and a superfamily of proteins with similarity to avirulence genes, has been found in the genome of the facultative parasites *P. ramorum* and *P. sojae* [44]. Meanwhile, compared to *Phytophthora* species, reduced RXLR gene number and fewer effector paralogs were found in *H. parasitica* [45].

### Conserved acetylated sites on histone N-terminal tails of oomycetes

**Fig. 6:**
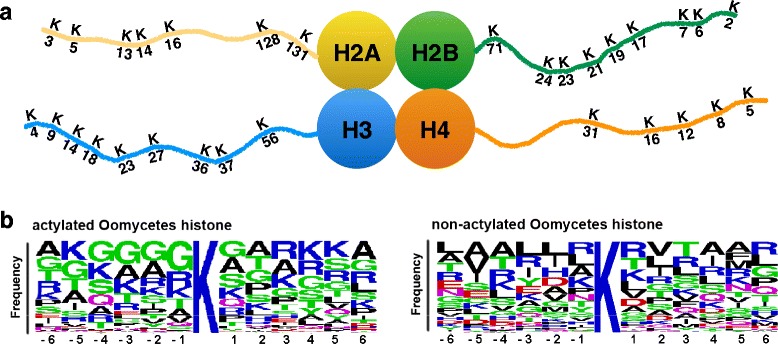
Conserved acetylated sites on histone N-termini shared by ten oomycetes species. **a** Computational predictions of histone acetylation sites on H2A, H2B, H3 and H4. The numbers indicate the positions of amino acids on the histone tails. **b** Frequency distribution of amino acids surrounding lysines in acetylated and non-acetylated histones. Frequency of amino acids (y axis) spanning positions −6 to 6 (x axis) showed differences in predicted acetylated lysines (698) (*left*) and non-acetylated lysines (321) (*right*) in the core histone protein. For sequences of lysines that are located close to each other, only one of them was picked up by analysis. Residues in polar amino acids are indicated in *green*, and the basic, acidic and hydrophobic amino acids are indicated in *blue*, *red* and *black*, respectively

To investigate conserved acetylated sites, the sequences of histone subunits in these ten oomycetes species were compared with reference species. Based on histone sequence alignments, we found that the histone subunits of oomycetes (H2A, H2B, H3, and H4) have highly conserved sequences, and many conserved acetylation sites were found on the histone tails of these oomycetes (Fig. 6a). The results suggested that 698 out of 1,019 identified lysines on histone subunits could be acetylated, such as H3K9, H3K14 and H3K56. The Fig. 6b illustrates the frequency distribution of amino acids surrounding lysine in the histone that could be acetylated.

### *HATs* and *HDACs* of *P. infestans* are expressed in stages of development, infection and responsive to stress

**Fig. 7:**
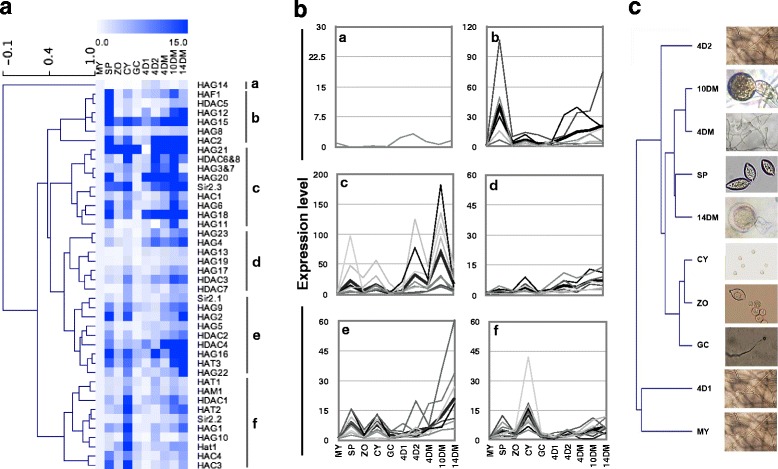
Clustering analysis of expression patterns of *HATs* and *HDACs* in *P. infestans* during development stages. The heat map **a** and the line charts **b** that generated by the clustering affinity search show the expression data of 33 *HATs* and 11 *HDACs* of *P. infestans* at different biological stages that detected with real-time qPCR. The color bar represents the log2 of expression fold changes, ranging from white (0.0) to blue (12.0). As the sequence identity within each of the two pairs of genes, *PifHAG3* and *PifHAG24*, *PifHDAC6* and *PifHDAC8*, is over than 97%, same primer was used for detecting the expression of each pair of genes. **c** Hierachical clustering tree of different samples. MY, mycelia; SP, sporangia; ZO, zoospores; CY, cysts; GC, germinated cysts; 4D1(or 2), the 4d old mycelia of A1 (or A2) isolate; respectively; 4DM, 10DM and 14DM, the 4d, 10d, and 14d old mycelia and oospores from junction areas between A1 and A2 isolates

In order to gain insight into the possible role of oomycetes HATs and HDACs, we examined the expression of predicted HATs and HDACs in ten developmental stages of *P. infestans.* As similar expression patterns were yielded from two biological replicates (Additional file 12), the results from the first experiment were used to draw the heatmap and construct the HCL tree. Our data showed that all 33 HATs and 11 HDACs predicted in *P. infestans* were exposed at ten developing stages with distinct patterns (Fig. 7) that distributed them into six groups (Fig. 7a and b). For example, genes in group b, including *PifHAG12* (XM_002900188.1)*, PifHAG15* (XM_002900191.1)*, PifHAF1* (XM_002908163.1)*,* and *PifHAC2* (XM_002997911.1), and *PifHDAC5* (XM_002998818.1)*,* were highly expressed at SP stage of asexual reproduction and at 4DM and 10DM stage of the sexual reproduction of *P. infestans*, while genes in Group e were up-regulated in SP, CY stages as well as in sexual reproduction stage. These data also demonstrated that the expression pattern of HATs and HDAC in the late oospore formation is different than those in the oogonia forming stage but more close to those in the sporangia forming stage (Fig. 7c, Additional file 12). Strikingly, some genes were specifically highly expressed in certain development stage, such as *PifHAG15* (XM_002900191.1) and *PifHAG18* (XM_002902054.1) were up-regulated more than 100 fold in SP stage, while *PifHAG20* (XM_002894902.1), *PifHAG21* (XM_002909614.1) and *PifHAG18* (XM_002902054.1) increased more than 100 fold at the late oogonia formation stage (10DM). The available microarray data and TPM at same development stages in previous study are consistent with the expression trends found in this study (Additional file 5) [65], except that less genes were detected in previous study due to the unavailable of genome sequence when those experiments were performed. Analysis of the ESTs data from previous study showed that the expression of 12 HATs and 11 HDACs genes, including *PifHAG5* (XM_002998432.1) and *PifHAG19* (XM_002903653.1) that hardly up-regulated during asexual and sexual reproduction stage, have activated in response to diverse environmental stresses (Additional file 5) including nutrition starvation and heat treatment.

Analysis of expression profiles of *P. infestans* during infection (Additional files 5 and 13) also revealed that the predicted 33 HATs and 11HDACs genes expressed differently during the infection. *PifHAG22* (XM_002907401.1), *PifHAM1* (XM_002908075.1)*, PifHDAC2* (XM_002904346.1)*,* and *PifHDAC3* (XM_002905236.1) were up-regulated in 2dpi, while 8 another HATs and HDACs including *PifHAG21* (XM_002909614.1), *PifHAT3* (XM_002895006.1), *PifHDAC4* (XM_002900466.1) and *PifSir2.3* (XM_002909246.1) were down-regulated in 2dpi (Additional files 5 and 13), demonstrating that the predicted HATs and HDACs are involved in the interaction between *P. infestans* and plant.

Previously, knowledge of epigenetic mechanisms in oomycetes was not available, except a few indirect evidences suggesting that histone acetylation may be involved in gene expression [8, 9, 74]. To our knowledge, this is the first study to identify and analyze the evolution and the express pattern of HATs and HDACs in oomycetes.

It is well known that a large genome dataset accelerates gene discovery in an organism. As gene manipulation techniques become available in several species of oomycetes, such as *P. infestans* and *P. sojae*, dataset resulted from this study will accelerate the investigation of epigenetic mechanisms in oomycetes. Further study of the epigenetic mechanisms in oomycetes will not only provide us with knowledge to manipulate the biology of these organisms, but will also benefit the understanding of epigenetic mechanisms in the many organisms in Stramenopiles.

## Conclusions

In this study, we have identified many HATs and HDACs in oomycetes, along with five histone acetyltransferase families (HAGs, HAFs, HAMs, HACs, and Hat1s), three putative novel HATs, and three classes (I, II and III) of histone deacetylases. We also reveal the structure of these *HATs* and *HDACs*. Phylogenetic analyses have suggested that most of these HATs and HDACs derived from distinct evolutionary ancestors, and most of them are closely related to those in marine algae and some other species in Stramenopiles. Phylogenetic based analysis unveiled the complex and distinct patterns of duplications and losses of HATs and HDACs in oomycetes. This study reveals the evolutionary dynamics that shaped the gene content of HATs and HDACs in oomycetes. In addition, sequence analysis revealed many conserved acetylation sites on histones and suggested that 698 out of 1,019 identified lysines could be acetylated. By analyzing the expression data of *HATs* and *HDACs* of *P. infestans* identified in this study, we are able to provide an overview of the specific biological stages that these genes involved, which give hints of their functions. Results of this study provide useful inputs to help explore the epigenetic mechanisms and the relationship between genomes and phenotypes of oomycetes.

## Additional files

Additional file 1: Gene information for HATs and HDACs of ten oomycetes species and the reference species used in this study. (XLS 301 kb)
Additional file 2: Orthologous and paralogous groups of HAGs and HDACs in oomycetes. (XLSX 291 kb)
Additional file 3: Primers for real-time qPCR used in this study. (DOC 156 kb)
Additional file 4: Sequences alignments of the amplified cDNA fragments and the targeted genes. (DOC 116 kb)
Additional file 5: EST, cDNA data and microarray expression values of *HATs* and *HDACs* of *Phytophthora infestans*. Data were from NCBI UniGene and GEO DataSets. (XLSX 33 kb)
Additional file 6: Phylogenetic tree of HAFs in oomycetes. A maximum-likelihood phylogenetic tree was constructed with sequences of HAF conserved domains from the species described in Fig. 2. Each domain was highlighted by one color and bootstrap values (≥50%) are shown near the tree nodes. (PDF 194 kb)
Additional file 7: Phylogenetic tree of HACs in oomycetes. A maximum-likelihood phylogenetic tree was constructed with sequences of HAC conserved domains from the species described in Fig. 2. All the predicted HACs of oomycetes were attributed to four clades according to the combination of conserved domains. These domains were highlighted by different colors and bootstrap values (≥50%) are shown near the tree nodes. (PDF 423 kb)
Additional file 8: Phylogenetic tree of HAMs in oomycetes. A maximum-likelihood phylogenetic tree was constructed with sequences of HAM conserved domains from the species described in Fig. 2. Different domains were highlighted by different colors and bootstrap values (≥50%) are shown near the tree nodes. (PDF 303 kb)
Additional file 9: Phylogenetic tree of Hat1s in oomycetes. A maximum-likelihood phylogenetic tree was constructed with sequences of Hat1 conserved domains from the species described in Fig. 2. Each Oomycetes species contained one Hat1 and they were attributed to 1 clade. Each domain was highlighted by one color and bootstrap values (≥50%) are shown near the tree nodes. (PDF 201 kb)
Additional file 10: Phylogenetic tree of putative novel HATs in oomycetes. A maximum-likelihood phylogenetic tree was constructed with sequences of the conserved domain AT-1 found in the species described in Fig. 2. Different domains were highlighted by different colors and bootstrap values (≥50%) are shown near the tree nodes. (PDF 189 kb)
Additional file 11: Sequence alignment of some Hpa2s in oomycetes. Bootstrap values (≥30%) and sequence identity are shown near the tree nodes. a Members of Hpa2 in the same species with high sequence identity aggregated in the same scaffold. b Members of Hpa2 in the same species with various sequence identities clustered in the same scaffold. (PDF 421 kb)
Additional file 12: Gene expression data of real-time qPCR in this study. (XLS 313 kb)
Additional file 13: Expression profiles of *HATs* and *HDACs* in *P. infestans* at various stages during infection. The heat map generated by microarray expression values of GSE14480 retrieved from GEO DataSets. The color bar represents the log_2_ of expression values, and the highest and lowest log2 signal value is 8.62 and 12.97, respectively. (PDF 62 kb)

## Abbreviations

acetyl CoA: Acetyl coenzyme A
DNMTs: DNA methyltransferases
Elp3: Elongator proteins 3
Gcn5: General control nonderepressible 5
HATs: Histone acetyltransferase
HDACs: Histone deacetylase
Hpa2: Heparanase 2
p300/CBP: CREB-binding protein
PTMs: Post-translational modifications
RPD3: Reduced potassium dependency 3
Sir2: Silent information regulator 2
TAF_II_250: TBP-associated factor subunits of TF_II_D
