# Supplementary material for: Diversity, evolution and expression profiles of histone acetyltransferases and deacetylases in oomycetes
Source: BMC Genomics. 2016 Nov 16;17:927. doi: 10.1186/s12864-016-3285-y (PMC5112689; doi:10.1186/s12864-016-3285-y)
Supplement: Additional file 3: — Primers for real-time qPCR used in this study. (DOC 156 kb) [file 12864_2016_3285_MOESM3_ESM.doc]

**Table S1.** The realtime qPCR primers used to amplify *HATs* and *HDACs* in *Phytophthora infestans* in this study

| Serial no. | Locus | Primer name | Primer sequence | GC content (%) | Melting temperature (℃) | Amplified length (bp) |
| --- | --- | --- | --- | --- | --- | --- |
| *PifHAG1* | PITG_20197.2 | HAT1-RT-F | ATCAAGGCAGCTTAAAGGCACAGCTCT | 48 | 57.4 | 259 |
|  |  | HAT1-RT-R | TAGTAGTTGGTGTCCGGCGTATTGTAGA | 46 | 57.5 |  |
| *PifHAG2* | PITG_08037.1 | HAG2F | GTTCGGCTGGAAAAAGCTCATTGAAGG | 48 | 59.7 | 121 |
|  |  | HAG2R | TACCAAGACTATACTCGCCGATTCAATGG | 45 | 60.1 |  |
| *PifHAG3^a^* | PITG_14481.2 | HAG3F | GACCGCGTAGACCTATTCATCCTG | 54 | 59.1 | 117 |
|  |  | HAG3R | ACACTGTTTTTGTCCCTCGTCCACG | 52 | 59.3 |  |
| *PifHAG4* | PITG_16695.2 | HAG4F | AGACGCGTCCTTGGGTACTATTCGA | 52 | 59.3 | 132 |
|  |  | HAG4R | AATTGGTCTGGTGTGATCGGATGCG | 52 | 59.3 |  |
| *PifHAG5* | PITG_06343.1 | HAG5F | CGATATTGAGAAGGGATACTACGCCG | 50 | 59.5 | 163 |
|  |  | HAG5R | AGAAGCCTCGTTCTCCTTGGCAG | 57 | 58.8 |  |
| *PifHAG6* | PITG_14006.2 | HAG6F | CTGGGGTATGTAAAGTACAACTACGTTGCT | 43 | 60.3 | 126 |
|  |  | HAG6R | TTGCGGTGTGTGGATGACAGCAGA | 54 | 59.1 |  |
| *PifHAT2* | PITG_06479.1 | HAG7F | ACGCGGATACGAAGGAGTGGATCT | 54 | 59.1 | 130 |
|  |  | HAG7R | TCCGGTAAATACTTCTCCGCGAGC | 54 | 59.1 |  |
| *PifHAG8* | PITG_11914.2 | HAG8F | AGACGGAGATCGCCAACAAGGGTG | 58 | 60.8 | 125 |
|  |  | HAG8R | CTGCAACCAAAGCTTAAGACGGTAGG | 50 | 59.5 |  |
| *PifHAG9* | PITG_22654.2 | HAG9F | TTCAAGACTCTGACACTCACGGCATC | 50 | 59.5 | 180 |
|  |  | HAG9R | AAGTTGTGGCGCGGGTTGGCAAGT | 58 | 60.8 |  |
| *PifHAG10* | PITG_03971.1 | HAG10F | ACGCCATCGAGGAACACGTACGTGA | 56 | 61.0 | 158 |
|  |  | HAG10R | GGCAGACTTGACCATGTAGCAGTGTT | 50 | 59.5 |  |
| *PifHAG11* | PITG_18300.2 | HAG11F | TGAAGAAACTCAACGTATCAGCGAAGGG | 46 | 59.9 | 134 |
|  |  | HAG11R | CAACTCGAATGCTAACCCACTCTTCC | 50 | 59.5 |  |
| *PifHAG12* | PITG_12865.2 | HAG12F | GCTCGAGAGTGTCATCAAGCAGGCT | 56 | 61.0 | 133 |
|  |  | HAG12R | GTTGCGCAGAACCTTGATTACTTCGAAG | 46 | 59.9 |  |
| *PifHAG13* | PITG_06396.1 | HAG13F | ACTGGCGCCATGAGGTTCTACAGAGAA | 52 | 61.3 | 117 |
|  |  | HAG13R | CTGTGTGACGCTTTTGCTCAGGATTTCG | 50 | 61.4 |  |
| *PifHAG14* | PITG_03972.1 | HAG14F | AGACTACACGCTCGGCCAAGATCG | 58 | 60.8 | 182 |
|  |  | HAG14R | GGTGCGTTGGTATCCACACTTTTCATAG | 46 | 59.9 |  |
| *PifHAG15* | PITG_12868.2 | HAG15F | ATATCTCCACGTGCAGACCAGCAACA | 50 | 59.5 | 139 |
|  |  | HAG15R | AGTGAGCTGTCTGCGTAGCACGTAG | 56 | 61.0 |  |
| *PifHAG16* | PITG_15429.2 | HAG16F | GGCTTCTATGTGAATTGCGGGTTCTC | 50 | 59.5 | 126 |
|  |  | HAG16R | TCCAACCTGAATCAATGGCGGTAGTCT | 48 | 59.7 |  |
| *PifHAG17* | PITG_02980.1 | HAG17F | TTTGAAGGCGTGATGCGGAAGCATCG | 54 | 61.1 | 183 |
|  |  | HAG17R | CTGTGCTTTCTTACCGGTATCTCCTTCA | 46 | 59.9 |  |
| *PifHAT3* | PITG_21166.2 | HAG18F | TCACCGTTAAACCACCTCCGCCTC | 58 | 60.8 | 157 |
|  |  | HAG18R | ATACCCAGAAGAACGGACCGGCGT | 58 | 60.8 |  |
| *PifHAG19* | PITG_08302.1 | HAG19F | AAAGCGGAGAAGGAGGGCGACAAGAT | 54 | 61.1 | 159 |
|  |  | HAG19R | CATCACGAGTGCTGTACGTGGCTCTAA | 52 | 61.3 |  |
| *PifHAG20* | PITG_21717.2 | HAG20F | GCCAAGCAGAACAAAATCAAGCGTGTC | 48 | 59.7 | 153 |
|  |  | HAG20R | GACGAACTTGACGAGGCGAAGATACT | 50 | 59.5 |  |
| *PifHAG21* | PITG_01143.1 | HAG21F | CGGACACGGGAAATCTGGGTGCTAT | 56 | 61.0 | 96 |
|  |  | HAG21R | CATGGTGACCTGGCTTGTAATAATCCG | 48 | 59.7 |  |
| *PifHAG18* | PITG_10274.1 | HAG22F | ACGCCTGCTTCTTCTTGGGGTTCGTT | 54 | 61.1 | 135 |
|  |  | HAG22R | GACGTCTGTTTGTTGACTCGAATTGCCAA | 45 | 60.1 |  |
| *PifHAT1* | PITG_11234.2 | HAG23F | TGTGGAGCGTTCAACGAGATCCGT | 54 | 59.1 | 114 |
|  |  | HAG23R | GCTGTGTTGCTTTTCACTTTCTACTTGCTC | 43 | 60.3 |  |
| *PifHAG7^a^* | PITG_14487.2 | HAG3F | GACCGCGTAGACCTATTCATCCTG | 54 | 59.1 | 117 |
|  |  | HAG3R | ACACTGTTTTTGTCCCTCGTCCACG | 52 | 59.3 |  |
| *PifHAG22* | PITG_02528.1 | HAG25F | TGGATGAGCTGCCAGTAGCAAGCAT | 52 | 59.3 | 152 |
|  |  | HAG25R | TCTTCTCCTGATCACAGCTGCAGTC | 52 | 59.3 |  |
| *PifHAG23* | PITG_01099.1 | HAG26F | AGAACGCATCGCATACGAAGAGCATG | 50 | 59.5 | 117 |
|  |  | HAG26R | TTGGACATGTAAGGTCCATCGATCTC | 46 | 58.0 |  |
| *PifHat1* | PITG_00186.1 | HAT6-RT-F | AAGCATTGACGACAGCAGACATCCACA | 48 | 57.4 | 277 |
|  |  | HAT6-RT-R | AACCCATTTTTTATCGCCATCTGCCGATAG | 43 | 57.7 |  |
| *PifHAM1* | PITG_01456.1 | HAT3-RT-F | GACATCTATCAAGAACGAGGACATTATCGC | 58 | 58.8 | 230 |
|  |  | HAT3-RT-R | CCTTGTCTGCTGTCATTGCTTTTAGCGA | 56 | 58.9 |  |
| *PifHAC1* | PITG_06355.1 | HAC1F | AGACCAACAAGACGCAGCTACTGCAT | 50 | 59.5 | 182 |
|  |  | HAC1R | CTTCAGCTTGTTTCTTCTTCTCCACCTC | 46 | 59.9 |  |
| *PifHAC2* | PITG_18027.2 | HAT5-RT-F | CTGCTTACTGGCGAGCGATCAATGTA | 46 | 57.5 | 269 |
|  |  | HAT5-RT-R | CATAGTTGAGTACTTTGCACGTCGGAAG | 48 | 57.4 |  |
| *PifHAC3* | PITG_07302.1 | HAT4-RT-F | ATGAAGGAGCTGATGCGTCACGGC | 58 | 58.8 | 278 |
|  |  | HAT4-RT-R | TCGCCTTGAGCTCGGCTCTGTTGTT | 56 | 58.9 |  |
| *PifHAC4* | PITG_08587.1 | HAT2-RT-F | AGCTCAACGTTTTCTGTCGCGAGTGC | 53 | 58.9 | 262 |
|  |  | HAT2-RT-R | CGCTCAACACGTTCACAAACGTTGAC | 50 | 57.3 |  |
| *PifHAF1* | PITG_01564.1 | HAF1F | AACGACACGAACGGCTCATTGGTTTTTAG | 45 | 60.1 | 145 |
|  |  | HAF1R | AAGAGCTTTCTTGGTGCTGATGTCGC | 50 | 59.5 |  |
| *PifHDAC1* | PITG_01897.1 | HDAC1F | CAGATGAGTCAAGCACCTCCCACG | 58 | 60.8 | 199 |
|  |  | HDAC1R | CTTCCTCCTGAGTAGAACCGTTGGCT | 54 | 61.1 |  |
| *PifHDAC2* | PITG_08237.1 | HDAC2F | TCAGTGCTGGAGGGAGGCTACAACTT | 54 | 61.1 | 233 |
|  |  | HDAC2R | TCAGTCTTACTGCGCTTCTTCGTCTGC | 52 | 61.3 |  |
| *PifHDAC3* | PITG_04499.1 | HDAC3F | ACTTGCCCGTGAGCAACATGGAAAAC | 50 | 59.5 | 271 |
|  |  | HDAC3R | TAAAATTCAACAGGATGACGGGGAGCGT | 46 | 59.9 |  |
| *PifHDAC4* | PITG_12962.2 | HDAC4F | CAGTGATTGAGGAATTGCTGGGGATTCA | 46 | 59.9 | 139 |
|  |  | HDAC4R | TGTAACTCAAGAACAGCGTCCACCACTT | 46 | 59.9 |  |
| *PifHDAC5* | PITG_05176.1 | HDAC5F | ACGACTTCTACTACTTCCTGAGTGAGGA | 46 | 59.9 | 187 |
|  |  | HDAC5R | TAATGGAAGTTGGAGATAGCACTCTTACGC | 43 | 60.3 |  |
| *PifHDAC6^b^* | PITG_21309.2 | HDAC6F | TATCGTTCCACAAATACGGCGACTTCTTC | 45 | 60.1 | 150 |
|  |  | HDAC6R | CTATCACTGGCTTGAATATGCTTTCGTAGC | 43 | 60.3 |  |
| *PifHDAC7* | PITG_15415.2 | HDAC7F | TATTAGAGAGAGATTCCCACGACTACCGA | 45 | 60.1 | 230 |
|  |  | HDAC7R | TCATGAGAGTCATATCGTCCCCCAGTT | 48 | 59.7 |  |
| *PifHDAC8^b^* | PITG_01911.1 | HDAC6F | TATCGTTCCACAAATACGGCGACTTCTTC | 45 | 60.1 | 150 |
|  |  | HDAC6R | CTATCACTGGCTTGAATATGCTTTCGTAGC | 43 | 60.3 |  |
| *PifSir2.1* | PITG_10164.1 | Sir2.1F | CGACGACGAAAGCAGCAGTTCTTCCA | 54 | 61.1 | 192 |
|  |  | Sir2.1R | AAGCTTGAGGCCCCTAGCAACTCGTT | 54 | 61.1 |  |
| *PifSir2.2* | PITG_06363.1 | Sir2.2F | AACTGGCAAGCACATCTATTGCTTTGGAGA | 43 | 60.3 | 173 |
|  |  | Sir2.2R | GCTCTGCTTCTGTGTCAATCTGGCGTT | 52 | 61.3 |  |
| *PifSir2.3* | PITG_00718.1 | Sir2.3F | GGAGAGCACGGAATCCGCACCAAAAA | 54 | 61.1 | 188 |
|  |  | Sir2.3R | ACGCCACTTTTTAGATGCAGATTGTCCAC | 45 | 60.1 |  |
| *PifEF1* | PITG_06722.1 | EF1F | ACTCCAAGAACGACCCTGCTAAGGCAACC | 55 | 64.3 | 241 |
|  |  | EF1R | TTCGACGGCTCGAGGATGACCATGCAG | 59 | 64.3 |  |

^a, b^ Gene followed with the same letters were detected with same primers as their sequence identity is over 97%.
