# Supplementary material for: Diversity, evolution and expression profiles of histone acetyltransferases and deacetylases in oomycetes
Source: BMC Genomics. 2016 Nov 16;17:927. doi: 10.1186/s12864-016-3285-y (PMC5112689; doi:10.1186/s12864-016-3285-y)
Supplement: Additional file 4: — Sequences alignments of the amplified cDNA fragments and the targeted genes. (DOC 116 kb) [file 12864_2016_3285_MOESM4_ESM.doc]

“Query” means sequence of DNA fragment amplified from cDNA using realtime qPCR primers in Additional file 3; “Sbjct” means sequence of the amplified fragment that hit the respective gene of *P. infestans* through genome BLAST. As Genbank has a policy that does not accept sequences shorter than 200 bp, the sequences longer than 200 bp have been submitted to NCBI GenBank.

> [***PifHAG1*** PITG_20197](http://www.genome.jp/dbget-bin/www_bget?pif:PITG_20197) **GenBank accession number: KX492573**

Query 24 CATCGGAGTGCATGGCCATTCCATGAGCCCGTGGACACCAGTGTCGTGGTTGACTACCTT 83

||||||||||||||||||||||||||||||||||||||||||||||||||||||||||||

Sbjct 1528 CATCGGAGTGCATGGCCATTCCATGAGCCCGTGGACACCAGTGTCGTGGTTGACTACCTT 1587

Query 84 GACCACATTAAGGAACCCATAGACCTCCAACTAATCAGCAAGCGCATCGACAGTGGAGCC 143

||||||||||||||||||||||||||||||||||||||||||||||||||||||||||||

Sbjct 1588 GACCACATTAAGGAACCCATAGACCTCCAACTAATCAGCAAGCGCATCGACAGTGGAGCC 1647

Query 144 TATATTTCCAAGGCTGCGTTCAAAGTAGACCTAGAGAAGATGTGCGACAACTGCATGCTC 203

||||||||||||||||||||||||||||||||||||||||||||||||||||||||||||

Sbjct 1648 TATATTTCCAAGGCTGCGTTCAAAGTAGACCTAGAGAAGATGTGCGACAACTGCATGCTC 1707

Query 204 TACAATACGCCGGACACCAACTACTA 228

||||||||||||||||||||||||||

Sbjct 1708 TACAATACGCCGGACACCAACTACTA 1733

> [***PifHAG2*** PITG_08037](http://www.genome.jp/dbget-bin/www_bget?pif:PITG_08037)

Query 31 TGATAAGCTTACCTTGTCCACAGCGTCAGCACCCATTGAATCGGCGAGTATAGTCTTGGTA 91

|||||||||||||||||||||||||||||||||||||||||||||||||||||||||||||

Sbjct 756 TGATAAGCTCACCCTGTCCACAGCGTCAGCACCCATTGAATCGGCGAGTATAGTCTTGGTA 816

> [***PifHAG3***  PITG_14481](http://www.genome.jp/dbget-bin/www_bget?pif:PITG_14481)

Query 15 CCTGCCAGCGCTGGTCGACAAGACAGGATTCTACGAAAAGTGCGGCTACAGCCGCATCGA 75

||||||||||||||||||||||||||||||||||||||||||||||||||||||||||||

Sbjct 321 CCTGCCAGCGCTGGTCGACAAGACAGGATTCTACGAAAAGTGCGGCTACAGCCGCATCGA 380

Query 76 CGACGAGATCTTCGTGGACG 95

||||||||||||||||||||

Sbjct 381 CGACGAGATCTTCGTGGACG 400

> [***PifHAG4*** PITG_16695](http://www.genome.jp/dbget-bin/www_bget?pif:PITG_16695)

Query 16 CGCTTTTGATATGAGGAAGGCACTGCCTCGAGACATCCACAAGAAGTCGATCATACCGTT 75

||||||||||||||||||||||||||||||||||||||||||||||||||||||||||||

Sbjct 435 CGCTTTTGATATGAGGAAGGCACTGCCTCGAGACATCCACAAGAAGTCGATCATACCGTT 494

Query 76 GCCGCATCCGATCACACCAGACCAATT 102

|||||||||||||||||||||||||||

Sbjct 495 GCCGCATCCGATCACACCAGACCAATT 521

> ***PifHAG5*** [PITG_06343](http://www.genome.jp/dbget-bin/www_bget?pif:PITG_06343)

Query 7 GACGCCTACGACATGCGTATGCCCTTTACGGACAAGTGCAACCAGGCGTTTTCGTCGCAG 64

||||||||||||||||||||||||||||||||||||||||||||||||||||||||||||

Sbjct 427 GACGCCTACGACATGCGTATGCCCTTTACGGACAAGTGCAACCAGGCGTTTTCGTCGCAG 486

Query 65 GTAAATCGCTTCAAGAAGGTGCTAGCAGAGAAAGATGCGGAGAAGGCTGCCAAGGAGAAC 124

||||||||||||||||||||||||||||||||||||||||||||||||||||||||||||

Sbjct 487 GTAAATCGCTTCAAGAAGGTGCTAGCAGAGAAAGATGCGGAGAAGGCTGCCAAGGAGAAC 546

Query 125 GAGGCTTCT 133

|||||||||

Sbjct 547 GAGGCTTCT 555

> ***PifHAG6*** [pif:PITG_14006](http://www.genome.jp/dbget-bin/www_bget?pif:PITG_14006)

Query 11 CTGGGGTATGTAAAGTACAACTACGTTGCTGCTTTTAACCAGGTCGAACTGGAGTTGGTC 71

||||||||||||||||||||||||||||||||||||||||||||||||||||||||||||

Sbjct 373 CTGGGGTATGTAAAGTACAACTACGTTGCTGCTTTTAACCAGGTCGAACTGGAGTTGGTC 432

Query 72 ACGAAGGACAAGCGAATGGAACTAGTGGACACCGTGTTGGAGTCTGCT 109

||||||||||||||||||||||||||||||||||||||||||||||||

Sbjct 433 ACGAAGGACAAGCGAATGGAACTAGTGGACACCGTGTTGGAGTCTGCT 480

> ***PifHAG7*** [pif:PITG_14487](http://www.genome.jp/dbget-bin/www_bget?pif:PITG_14487)

Query 7 CCTGCCAGCGCTGGTCGACAAGACAGGATTCTACGAAAAGTGCGGCTACAGCCGCATCGA 66

||||||||||||||||||||||||||||||||||||||||||||||||||||||||||||

Sbjct 246 CCTGCCAGCGCTGGTCGACAAGACAGGATTCTACGAAAAGTGCGGCTACAGCCGCATCGA 305

Query 67 CGACGAGATCTTCGTGGACG 86

||||||||||||||||||||

Sbjct 306 CGACGAGATCTTCGTGGACG 325

> [***PifHAG8*** PITG_11914](http://www.genome.jp/dbget-bin/www_bget?pif:PITG_11914)

Query 7 CTATATGAGAACCGGGGCTTTGTGCGTGACGAGCGGTTGGTCAAGTACTATTTAAACGGG 66

||||||||||||||||||||||||||||||||||||||||||||||||||||||||||||

Sbjct 445 CTATATGAGAACCTGGGCTTTGTGCGTGACGAGCGGTTGGTCAAGTACTATTTAAACGGG 504

Query 67 GGTGACGCCTACCGTCTTAAGCTTTGGTTGCAG 99

|||||||||||||||||||||||||||||||||

Sbjct 505 GGTGACGCCTACCGTCTTAAGCTTTGGTTGCAG 537

> [***PifHAG9*** PITG_22654](http://www.genome.jp/dbget-bin/www_bget?pif:PITG_22654)

Query 13 AGACGTCGGCCGTTAAGTTTTACACTTCATTTGGCTTTCAAATTGGGGAAAACATCATGT 71

||||||||||||||||||||||||||||||||||||||||||||||||||||||||||||

Sbjct 338 AGACGTCGGCCGTTAAGTTTTACACTTCATTTGGCTTTCAAATTGGGGAAAACATCATGT 397

Query 72 TTTGGGAAAACCCTTCCCACGAGGTATTCTGGATGACCAAGACGCTATCGCCTCTGCCTA 131

||||||||||||||||||||||||||||||||||||||||||||||||||||||||||||

Sbjct 398 TTTGGGAAAACCCTTCCCACGAGGTATTCTGGATGACCAAGACGCTATCGCCTCTGCCTA 457

Query 132 CTTGCCAACCCGCGCCACAACTT 154

|||||||||||||||||||||||

Sbjct 458 CTTGCCAACCCGCGCCACAACTT 480

> [***PifHAG10*** PITG_03971](http://www.genome.jp/dbget-bin/www_bget?pif:PITG_03971)

Query 12 TGTTCGTGCTGTCCTCACAGTACGGACGCAAGGGGTTCTACGAGAAGTGCGGTTACCACT 69

||||||||||||||||||||||||||||||||||||||||||||||||||||||||||||

Sbjct 326 TGTTCGTGCTGTCCTCACAGTACGGACGCAAGGGGTTCTACGAGAAGTGCGGTTACCACT 385

Query 70 GCCCAAGCGACGAGATTTACCTGGAGGAAGGAGCAAAACACTGCTACATGGTCAAGTCTGCC 131

||||||||||||||||||||||||||||||||||||||||||||||||||||||||||||||

Sbjct 386 GCCCAAGCGACGAGATTTACCTGGAGGAAGGAGCAAAACACTGCTACATGGTCAAGTCTGCC 447

> [***PifHAG11*** PITG_18300](http://www.genome.jp/dbget-bin/www_bget?pif:PITG_18300)

Query 6 GGCGACTCGAGTGGTCGTGTCTGCGCTGGAACGAAGGAGCACTCAAGTTCTACGAGCGTA 64

||||||||||||||||||||||||||||||||||||||||||||||||||||||||||||

Sbjct 404 GGCGACTCGAGTGGTCGTGTCTGCGCTGGAACGAAGGAGCACTCAAGTTCTACGAGCGTA 463

Query 65 TTGGAGGCACACGCATGGAAGAGTGGGTTAGCATTCGAGTTG 106

||||||||||||||||||||||||||||||||||||||||||

Sbjct 464 TTGGAGGCACACGCATGGAAGAGTGGGTTAGCATTCGAGTTG 505

> [***PifHAG12*** PITG_12865](http://www.genome.jp/dbget-bin/www_bget?pif:PITG_12865)

Query 10 AGCTGGTCTACGTGTACTTGCACGTGCTGAGCTCCAATACCGCCGCGCATCGCTTCTACT 69

||||||||||||||||||||||||||||||||||||||||||||||||||||||||||||

Sbjct 266 AGCTGGTCTACGTGTACTTGCACGTGCTGAGCTCCAATACCGCCGCGCATCGCTTCTACT 325

Query 70 TAACTCACGGCTTCGAAGTAATCAAGGTTCTGCGCAAC 107

||||||||||||||||||||||||||||||||||||||

Sbjct 326 TAACTCACGGCTTCGAAGTAATCAAGGTTCTGCGCAAC 363

> [***PifHAG13*** PITG_06396](http://www.genome.jp/dbget-bin/www_bget?pif:PITG_06396)

Query 19 TGAGACGTCGCCGAGTGCGTGTGGAGACGACTCGCAAGACTACGAAATCCTGAGCAAAAG 78

||||||||||||||||||||||||||||||||||||||||||||||||||||||||||||

Sbjct 471 TGAGACGTCGCCGAGTGCGTGTGGAGACGACTCGCAAGACTACGAAATCCTGAGCAAAAG 530

Query 79 CGTCACACAG 88

||||||||||

Sbjct 531 CGTCACACAG 540

> [***PifHAG14*** PITG_03972](http://www.genome.jp/dbget-bin/www_bget?pif:PITG_03972)

Query 5 CGTGGCAGTGCTCCAGGAATGCCGCGGCAAGAGCTACGGCGTCGCGTTGATGGAAGGCAT 60

||||||||||||||||||||||||||||||||||||||||||||||||||||||||||||

Sbjct 234 CGTGGCAGTGCTCCAGGAATGCCGCGGCAAGAGCTACGGCGTCGCGTTGATGGAAGGCAT 293

Query 61 GGAGCGCCTCGTTAAAGCTTTCGGCACAGCTCGACAAGAAGGGATTCTATGAAAAGTGTG 120

||||||||||||||||||||||||||||||||||||||||||||||||||||||||||||

Sbjct 294 GGAGCGCCTCGTTAAAGCTTTCGGCACAGCTCGACAAGAAGGGATTCTATGAAAAGTGTG 373

Query 121 GATACCAACGCACC 134

||||||||||||||

Sbjct 374 GATACCAACGCACC 387

> [***PifHAG15*** PITG_12868](http://www.genome.jp/dbget-bin/www_bget?pif:PITG_12868)

Query 9 GGCGGCTCGATGTGTTTGTAGTAGTCGCGTAGGATCTGTGTGGCCTCGAAGCCATGTGAG 68

||||||||||||||||||||||||||||||||||||||||||||||||||||||||||||

Sbjct 482 GGCGGCTCGATGTGTTTGTAGTAGTCGCGTAGGATCTGTGTGGCCTCGAAGCCATGTGAG 423

Query 69 CGATAGAAACGTAGGGCCGCGCTGTTGCTGGTCTGCACGTGGAGATATA 117

|||||||||||||||||||||||||||||||||||||||||||||||||

Sbjct 422 CGATAGAAACGTAGGGCCGCGCTGTTGCTGGTCTGCACGTGGAGATATA 374

> [***PifHAG16*** PITG_15429](http://www.genome.jp/dbget-bin/www_bget?pif:PITG_15429)

Query 17 CAGCGACAGTTCGAACCACGGATCTTGGCCGTGTACAACCGGCGACAGTCGCGTCACGGA 74

||||||||||||||||||||||||||||||||||||||||||||||||||||||||||||

Sbjct 258 CAGCGACAGTTCGAACCACGGATCTTGGCCGTGTACAACCGGCGACAGTCGCGTCACGGA 199

Query 75 GAACCCGCAATTCACATAGAAGCCA 99

|||||||||||||||||||||||||

Sbjct 198 GAACCCGCAATTCACATAGAAGCCA 174

>***PifHAG17***  [PITG_02980](http://www.genome.jp/dbget-bin/www_bget?pif:PITG_02980)

Query 11 ATCGGGACACAGTCGTCTTTGCTGCTATCAACAGCGACTGGGTGGTCATGGAGGAACACT 69

||||||||||||||||||||||||||||||||||||||||||||||||||||||||||||

Sbjct 746 ATCGGGACACAGTCGTCTTTGCTGCTATCAACAGCGACTGGGTGGTCATGGAGGAACACT 805

Query 70 TGCAACATAAACTTCATCAAGCTTTGCAGAAGGAGGAATCAAGCGCACCAGCTGAAGGAG 129

||||||||||||||||||||||||||||||||||||||||||||||||||||||||||||

Sbjct 806 TGCAACATAAACTTCATCAAGCTTTGCAGAAGGAGGAATCAAGCGCACCAGCTGAAGGAG 865

Query 130 ATACCGGTAAGAAAGCACAG 149

||||||||||||||||||||

Sbjct 866 ATACCGGTAAGAAAGCACAG 885

> [***PifHAG18*** PITG_10274](http://www.genome.jp/dbget-bin/www_bget?pif:PITG_10274)

Query 12 GACGTTCGATTCCTTACTCCTTCGCCCAGCTTTCAAGTTCCACCATGAGCTTCCGTCCTA 70

||||||||||||||||||||||||||||||||||||||||||||||||||||||||||||

Sbjct 576 GACGTTCGATTCCTTACTCCTTCGCCCAGCTTTCAAGTTCCACCATGAGCTTCCGTCCTA 635

Query 71 CCCCCATGCGTTGGCAATTCGAGTCAACAAACAGACGTC 109

|||||||||||||||||||||||||||||||||||||||

Sbjct 636 CCCCCATGCGTTGGCAATTCGAGTCAACAAACAGACGTC 674

> [***PifHAG19*** PITG_08302](http://www.genome.jp/dbget-bin/www_bget?pif:PITG_08302)

Query 16 TGTTCATGCCGGAAATGACGAAGCGATGGCGTTCTACAAGGCACTGGGGTTCGTTGAAAA 73

||||||||||||||||||||||||||||||||||||||||||||||||||||||||||||

Sbjct 345 TGTTCATGCCGGAAATGACGAAGCGATGGCGTTCTACAAGGCACTGGGGTTCGTTGAAAA 404

Query 74 AGCCCGTGTCGAAGACTACTATCGCCACTTAGAGCCACGTACAGCACTCGTGATG 128

|||||||||||||||||||||||||||||||||||||||||||||||||||||||

Sbjct 405 AGCCCGTGTCGAAGACTACTATCGCCACTTAGAGCCACGTACAGCACTCGTGATG 459

> [***PifHAG20*** PITG_21717](http://www.genome.jp/dbget-bin/www_bget?pif:PITG_21717)

Query 11 TGGCGCAAACAGTGATGGACCAAAAGCATTTTACACATCGCTGGGGTATACCAAGCTGGA 69

||||||||||||||||||||||||||||||||||||||||||||||||||||||||||||

Sbjct 408 TGGCGCAAACAGTGATGGACCAAAAGCATTTTACACATCGCTGGGGTATACCAAGCTGGA 467

Query 70 TGAGATCCTGTACAACTGGACGAATCCCCAGTATCTTCGCCTCGTCAAGTTCGTC 124

|||||||||||||||||||||||||||||||||||||||||||||||||||||||

Sbjct 468 TGAGATCCTGTACAACTGGACGAATCCCCAGTATCTTCGCCTCGTCAAGTTCGTC 522

> [***PifHAG21*** PITG_01143](http://www.genome.jp/dbget-bin/www_bget?pif:PITG_01143)

Query 13 CAGGGCTTCACGCGCACTACGGTCCGCGCGGATTATTACAAGCCAGGTCACCA 64

|||||||||||||||||||||||||||||||||||||||||||||||||||||

Sbjct 307 CAGGGCTTCACGCGCACTACGGTCCGCGCGGATTATTACAAGCCAGGTCACCA 359

> [***PifHAG22*** PITG_02528](http://www.genome.jp/dbget-bin/www_bget?pif:PITG_02528)

Query 10 CGCGTTCGTTAACGAGAACTTGAGTCGCGATTTCTCAACCACCCAGGTCCGTACGGCTAT 67

||||||||||||||||||||||||||||||||||||||||||||||||||||||||||||

Sbjct 1941 CGCGTTCGTTAACGAGAACTTGAGTCGCGATTTCTCAACCACCCAGGTCCGTACGGCTAT 2000

Query 68 CGTGAGCGCTGTTGTCGTTAGTGCAGTGACTGCAGCTGTGATCAGGAGAAGA 119

||||||||||||||||||||||||||||||||||||||||||||||||||||

Sbjct 2001 CGTGAGCGCTGTTGTCGTTAGTGCAGTGACTGCAGCTGTGATCAGGAGAAGA 2052

> [***PifHAG23*** PITG_01099](http://www.genome.jp/dbget-bin/www_bget?pif:PITG_01099)

Query 10 CTCGTGGTTATCGCTGGCGTCGGCACTCGCAACTACTACCGCAAACTGGGCTATGAGATC 69

||||||||||||||||||||||||||||||||||||||||||||||||||||||||||||

Sbjct 1537 CTCGTGGTTATCGCTGGCGTCGGCACTCGCAACTACTACCGCAAACTGGGCTATGAGATC 1596

Query 70 GATGGACCTTACATGTCCAAA 90

|||||||||||||||||||||

Sbjct 1597 GATGGACCTTACATGTCCAAA 1617

> [***PifHAC1*** PITG_06355](http://www.genome.jp/dbget-bin/www_bget?pif:PITG_06355)

Query 14 TGTCTGGGAACTGCGCAAAGCGTGCGAAGGAGCTGAGTGAGCGTATCTTGCCGCTCAACG 71

||||||||||||||||||||||||||||||||||||||||||||||||||||||||||||

Sbjct 2585 TGTCTGGGAACTGCGCAAAGCGTGCGAAGGAGCTGAGTGAGCGTATCTTGCCGCTCAACG 2644

Query 72 AGAAGGCGGCAGCGGCTGAGCTGGCGGAGCTGGATCTGCAAGAGATCAAG 121

||||||||||||||||||||||||||||||||||||||||||||||||||

Sbjct 2645 AGAAGGCGGCAGCGGCTGAGCTGGCGGAGCTGGATCTGCAAGAGATCAAG 2694

> ***PifHAC2***  [PITG_18027](http://www.genome.jp/dbget-bin/www_bget?pif:PITG_18027) **GenBank accession number: KX492574**

Query 9 ACTACTGCAGGCAGTGCCAGGGTGCAGTAGCGGAGGCAATCGCTCCTGCTTCTGGACA 68

||||||||||||||||||||||||||||||||||||||||||||||||||||||||||

Sbjct 2345 ACTACTGCAGCAAGTGCCAGGGTGCAGTAGCGGAGGCAATCGCTCCTGCTTCTGGACA 2402

Query 69 GAAGGCGGTGCTTACTAAAGTTGCTCCGGCCAACTTCGCTGAAGCCTGCAGCAATAGCAA 128

||||||||||||||||||||||||||||||||||||||||||||||||||||||||||||

Sbjct 2403 GAAGGCGGTGCTTACTAAAGTTGCTCCGGCCAACTTCGCTGAAGCCTGCAGCAATAGCAA 2462

Query 129 GACGGAGGAACTGGAGATGTCGTGCCCGTTCCTTGACTGCCGCCCCAATATGCTAAAGAA 188

||||||||||||||||||||||||||||||||||||||||||||||||||||||||||||

Sbjct 2463 GACGGAGGAACTGGAGATGTCGTGCCCGTTCCTTGACTGCCGCCCCAATATGCTAAAGAA 2522

Query 189 TTGTGAAGAGCACCACTACCAGTTCGACAGCTTCCGACGTGCAAAGTACTCAACTATGAG 248

||||||||||||||||||||||||||||||||||||||||||||||||||||||||||||

Sbjct 2523 TTGTGAAGAGCACCACTACCAGTTCGACAGCTTCCGACGTGCAAAGTACTCAACTATGAG 2582

> ***PifHAC3*** PITG_07302 **GenBank accession number: KX492575**

Query 29 GGCTGCACGATCTGTCGGCGTGTGTGGGCGCTACTGCAGCTCCACGCTCGGCAGTGTCGC 88

||||||||||||||||||||||||||||||||||||||||||||||||||||||||||||

Sbjct 6637 GGCTGCACGATCTGTCGGCGTGTGTGGGCGCTACTGCAGCTCCACGCTCGGCAGTGTCGC 6696

Query 89 CAATATGAGTGCAAGGTGCCGCGCTGTCACGACCTGCGGGAGCATGTGCGCAAGCTGCAG 148

||||||||||||||||||||||||||||||||||||||||||||||||||||||||||||

Sbjct 6697 CAATATGAGTGCAAGGTGCCGCGCTGTCACGACCTGCGGGAGCATGTGCGCAAGCTGCAG 6756

Query 149 TTACAGCAGCAACTCATGGACGACCGTCGTCGCGCTGCTGTCACGCAACAGTATCGCCAG 208

||||||||||||||||||||||||||||||||||||||||||||||||||||||||||||

Sbjct 6757 TTACAGCAGCAACTCATGGACGACCGTCGTCGCGCTGCTGTCACGCAACAGTATCGCCAG 6816

Query 209 ATGCAGAACGAGCGACAACAGGAACAACAGAGCCGAGCTCAAGGCGA 255

|||||||||||||||||||||||||||||||||||||||||||||||

Sbjct 6817 ATGCAGAACGAGCGACAACAGGAACAACAGAGCCGAGCTCAAGGCGA 6863

**>** [***PifHAC4*** PITG_08587](http://www.genome.jp/dbget-bin/www_bget?pif:PITG_08587) **GenBank accession number: KX492576**

Query 17 GCGCAGACGTTTGGTCTTGTCGGACGTGCGTACGCTTCGCTCTGTGCGATACCTGCTTTC 76

||||||||||||||||||||||||||||||||||||||||||||||||||||||||||||

Sbjct 3323 GCGCAGACGTTTGGTCTTGTCGGACGTGCGTACGCTTCGCTCTGTGCGATACCTGCTTTC 3382

Query 77 GACGTCAGGGCAGTGAACACCCACATCAGCTTTTCTTTGGATCCGCCGCACTGACGGTGC 136

||||||||||||||||||||||||||||||||||||||||||||||||||||||||||||

Sbjct 3383 GACGTCAGGGCAGTGAACACCCACATCAGCTTTTCTTTGGATCCGCCGCACTGACGGTGC 3442

Query 137 CGCCTCGAGTGCAGCGGATGTCGGAGCCACAGCAGCATGTTGAGCCTCAGACGTCGGTGC 196

||||||||||||||||||||||||||||||||||||||||||||||||||||||||||||

Sbjct 3443 CGCCTCGAGTGCAGCGGATGTCGGAGCCACAGCAGCATGTTGAGCCTCAGACGTCGGTGC 3502

Query 197 AACTGCCGCCCACCGTCAACGTTTGTGAACGTGTTGAGCG 236

||||||||||||||||||||||||||||||||||||||||

Sbjct 3503 AACTGCCGCCCACCGTCAACGTTTGTGAACGTGTTGAGCG 3542

> [***PifHat1*** PITG_00186](http://www.genome.jp/dbget-bin/www_bget?pif:PITG_00186) **GenBank accession number: KX492577**

Query 13 AGCTCAAAATCACACAAAAGCAGGTGCAGACCTGCTACGAGACGCGCAAGTTCGCGCTCG 71

||||||||||||||||||||||||||||||||||||||||||||||||||||||||||||

Sbjct 1007 AGCTCAAAATCACACAAAAGCAGGTGCAGACCTGCTACGAGACGCGCAAGTTCGCGCTCG 1066

Query 72 TGGACCCGAGCGACGAGGCGCAACTCAAGAAATTTCGGCTTGAGGTCAAGAAGCGGCTGT 131

||||||||||||||||||||||||||||||||||||||||||||||||||||||||||||

Sbjct 1067 TGGACCCGAGCGACGAGGCGCAACTCAAGAAATTTCGGCTTGAGGTCAAGAAGCGGCTGT 1126

Query 132 TTCGCTTGCATACTGAGGAGCTGGACGGCATGGGCGCCGACCGCCGCAAAGCTTTCCTCG 191

||||||||||||||||||||||||||||||||||||||||||||||||||||||||||||

Sbjct 1127 TTCGCTTGCATACTGAGGAGCTGGACGGCATGGGCGCCGACCGCCGCAAAGCTTTCCTCG 1186

Query 192 AGGCCGAGTATCAGAAACTGGAAGAGCACTATCGGCAGATGGCGATAAAAAATGGGTT 249

||||||||||||||||||||||||||||||||||||||||||||||||||||||||||

Sbjct 1187 AGGCCGAGTATCAGAAACTGGAAGAGCACTATCGGCAGATGGCGATAAAAAATGGGTT 1244

> [***PifHAF1*** PITG_01564](http://www.genome.jp/dbget-bin/www_bget?pif:PITG_01564)

Query 28 ATGACTTGCCTCCATCGAAGCTGGCCAAGATCGACCTGAGGCGCAAGTCCAAAGTCTAC 87

|||||||||||||||||||||||||||||||||||||||||||||||||||||||||||

Sbjct 1583 ATGACTTGCCTCCATCGAAGCTGGCCAAGATCGACCTGAGGCGCAAGTCCAAAGTCTAC 1641

Query 88 ATTTGCGACATCAGCACCAAGAAAGCTCTT 117

||||||||||||||||||||||||||||||

Sbjct 1642 ATTTGCGACATCAGCACCAAGAAAGCTCTT 1671

> ***PifHAM1***  [PITG_01456](http://www.genome.jp/dbget-bin/www_bget?pif:PITG_01456) **GenBank accession number: KX492578**

Query 21 GAAGTATCGATCATGGAGCTTACTCGGATGACATCTATCAAGAACGAGGACATTATCGCG 80

||||||||||||||||||||||||||||||||||||||||||||||||||||||||||||

Sbjct 1141 GAAGTATCGATCATGGAGCTTACTCGGATGACATCTATCAAGAACGAGGACATTATCGCG 1200

Query 81 ACGCTGCAGCACCTCAACATGATCAAGTACCTAGGCGGACAGTACGTGTACGTGGTTCCA 140

||||||||||||||||||||||||||||||||||||||||||||||||||||||||||||

Sbjct 1201 ACGCTGCAGCACCTCAACATGATCAAGTACCTAGGCGGACAGTACGTGTACGTGGTTCCA 1260

Query 141 AGACAGATCGTGGACGCGCACCTGACAAAGCTGACGAAGAAGGGTCCACAGGTCGTTCCG 200

||||||||||||||||||||||||||||||||||||||||||||||||||||||||||||

Sbjct 1261 AGACAGATCGTGGACGCGCACCTGACAAAGCTGACGAAGAAGGGTCCACAGGTCGTTCCG 1320

Query 201 GAGAAGCTGCACTGGGCTCCGCTGCAC 227

|||||||||||||||||||||||||||

Sbjct 1321 GAGAAGCTGCACTGGGCTCCGCTGCAC 1347

> [***PifHAT1*** PITG_11234](http://www.genome.jp/dbget-bin/www_bget?pif:PITG_11234)

Query 14 ATGGAAGGCGCTCAGCAACTTTACCGCTCACTCGGATACGTTGAGCAAGTAGAAAGTGAA 71

||||||||||||||||||||||||||||||||||||||||||||||||||||||||||||

Sbjct 1084 ATGGAAGGCGCTCAGCAACTTTACCGCTCACTCGGATACGTTGAGCAAGTAGAAAGTGAA 1143

Query 72 AAGCAACACAGC 83

||||||||||||

Sbjct 1144 AAGCAACACAGC 1155

> [***PifHAT2*** PITG_06479](http://www.genome.jp/dbget-bin/www_bget?pif:PITG_06479)

Query 16 TAGCTTGTGTGTCAGAGGAAGTAGTGCAGAAGGTCAGCGTGAGGGATGAACTACCGTA 75

||||||||||||||||||||||||||||||||||||||||||||||||||||||||||

Sbjct 1556 TAGCTTGTGTGTCAGAGGAAGTAGTGCAGAAGGTCAGCGTGAGGGATGAACTACCGTA 1613

Query 76 CTTCATGCAGCTCGCGGAGAAGTATTTACCGGA 108

|||||||||||||||||||||||||||||||||

Sbjct 1614 CTTCATGCAGCTCGCGGAGAAGTATTTACCGGA 1646

> [***PifHAT3*** PITG_21166](http://www.genome.jp/dbget-bin/www_bget?pif:PITG_21166)

Query 11 AGATGACATCCGTCGTGTCGCTGCGATGGAAGCTGCTTCTTATCCCGCTGACGAAGCGGC 69

||||||||||||||||||||||||||||||||||||||||||||||||||||||||||||

Sbjct 45 AGATGACATCCGTCGTGTCGCTGCGATGGAAGCTGCTTCTTATCCCGCTGACGAAGCGGC 104

Query 70 TACTGAGTCGGGCATACGATTCCGACAGAAAAACGCCGGTCCGTTCTTCTGGGTAT 125

||||||||||||||||||||||||||||||||||||||||||||||||||||||||

Sbjct 105 TACTGAGTCGGGCATACGATTCCGACAGAAAAACGCCGGTCCGTTCTTCTGGGTAT 160

> [***PifHDAC1*** PITG_01897](http://www.genome.jp/dbget-bin/www_bget?pif:PITG_01897)

Query 33 CATTCCCGCGCTGGTCCTTGTCGTCTCTATAGAATTCCGCCTCGTGCTGTCGCTTGCCGT 92

||||||||||||||||||||||||||||||||||||||||||||||||||||||||||||

Sbjct 1282 CATTCCCGCGCTGGTCCTTGTCGTCTCTATAGAATTCCGCCTCGTGCTGTCGCTTGCCGT 1223

Query 93 CATTGTCGGTTCGTGCGTCAGGGTCTGCTGCGTCTTCGTCCTCTTCCCGTAACATGTGCG 152

||||||||||||||||||||||||||||||||||||||||||||||||||||||||||||

Sbjct 1222 CATTGTCGGTTCGTGCGTCAGGGTCTGCTGCGTCTTCGTCCTCTTCCCGTAACATGTGCG 1163

Query 153 TGGGAGGTGCTTGACTCATCTGA 175

|||||||||||||||||||||||

Sbjct 1162 TGGGAGGTGCTTGACTCATCTGA 1140

> [***PifHDAC2*** PITG_08237](http://www.genome.jp/dbget-bin/www_bget?pif:PITG_08237) **GenBank accession number: KX492579**

Query 14 ATCTCGCCCTTCGCACGTAGCGTGGCAGCCCACACACGTGCGCTTATCAACCCAGCACAA 72

||||||||||||||||||||||||||||||||||||||||||||||||||||||||||||

Sbjct 2302 ATCTCGCCCTTCGCACGTAGCGTGGCAGCCCACACACGTGCGCTTATCAACCCAGCACAA 2361

Query 73 GAGCTCTGGGACGAGGACGAGATCGCCAAGGAGGCTGCACACGAGCAGGCATTGCTGGCT 132

||||||||||||||||||||||||||||||||||||||||||||||||||||||||||||

Sbjct 2362 GAGCTCTGGGACGAGGACGAGATCGCCAAGGAGGCTGCACACGAGCAGGCATTGCTGGCT 2421

Query 133 AATTATTTAGAGCCAACTGCAGCAACGTCTGCGGGCGTGACGATGCTGCAGACGAAGAAG 192

||||||||||||||||||||||||||||||||||||||||||||||||||||||||||||

Sbjct 2422 AATTATTTAGAGCCAACTGCAGCAACGTCTGCGGGCGTGACGATGCTGCAGACGAAGAAG 2481

Query 193 CGCAGTAAGACTGA 206

||||||||||||||

Sbjct 2482 CGCAGTAAGACTGA 2495

> [***PifHDAC3*** PITG_04499](http://www.genome.jp/dbget-bin/www_bget?pif:PITG_04499) **GenBank accession number: KX492580**

Query 18 GGACGAAATTAAGCGTCAGATCCATGAGAACCTCCGCCAAATCGAACCTGTTCCCAGTGT 76

||||||||||||||||||||||||||||||||||||||||||||||||||||||||||||

Sbjct 1113 GGACGAAATTAAGCGTCAGATCCATGAGAACCTCCGCCAAATCGAACCTGTTCCCAGTGT 1172

Query 77 GCCTTTCACAGTCGCACCACCGTCAGCTCGAATTCAAGAGGAGAAGGAAGCTGCCGCGCG 136

||||||||||||||||||||||||||||||||||||||||||||||||||||||||||||

Sbjct 1173 GCCTTTCACAGTCGCACCACCGTCAGCTCGAATTCAAGAGGAGAAGGAAGCTGCCGCGCG 1232

Query 137 CGACCGTGAGGATGACGATCAGCACATGATGGATGTGTCGGGCGAACGTCAAGACCAACA 196

||||||||||||||||||||||||||||||||||||||||||||||||||||||||||||

Sbjct 1233 CGACCGTGAGGATGACGATCAGCACATGATGGATGTGTCGGGCGAACGTCAAGACCAACA 1292

Query 197 AGAACTCAAGCAGAGCGACGCTCCCCGTCATCCTGTTGAATTTTA 241

|||||||||||||||||||||||||||||||||||||||||||||

Sbjct 1293 AGAACTCAAGCAGAGCGACGCTCCCCGTCATCCTGTTGAATTTTA 1337

> ***PifHDAC4*** [PITG_12962](http://www.genome.jp/dbget-bin/www_bget?pif:PITG_12962)

Query 11 GAGGAATTGCTGGGGATTCAAGACGTTGACAAGCAGATAAAGGATCCATTTCTAAGCGAA 70

||||||||||||||||||||||||||||||||||||||||||||||||||||||||||||

Sbjct 961 GAGGAATTGCTGGGGATTCAAGACGTTGACAAGCAGATAAAGGATCCATTTCTAAGCGAA 1020

Query 71 GTGGAGCGGTGGGAGTACCAAGAGCTGCAAGAACACCAGAAGAAAGTGGTGGACGCTGTT 130

||||||||||||||||||||||||||||||||||||||||||||||||||||||||||||

Sbjct 1021 GTGGAGCGGTGGGAGTACCAAGAGCTGCAAGAACACCAGAAGAAAGTGGTGGACGCTGTT 1080

> [***PifHDAC5*** PITG_05176](http://www.genome.jp/dbget-bin/www_bget?pif:PITG_05176)

Query 11 CTGGATCACAGAGCAAATCACTGCTGTTGCTGAAGATTGCTGTGACGGGCGCATTATTTC 68

||||||||||||||||||||||||||||||||||||||||||||||||||||||||||||

Sbjct 1782 CTGGATCACAGAGCAAATCACTGCTGTTGCTGAAGATTGCTGTGACGGGCGCATTATTTC 1841

Query 69 GGTGCTCGAGGGCGGCTACAATGTTGTGCCTTCCAAGTGCCAGCGTCCGCAACCTAAGGC 128

||||||||||||||||||||||||||||||||||||||||||||||||||||||||||||

Sbjct 1842 GGTGCTCGAGGGCGGCTACAATGTTGTGCCTTCCAAGTGCCAGCGTCCGCAACCTAAGGC 1901

Query 129 GCGTAAGAGTGCTATCTCCAACTTCCATTA 158

||||||||||||||||||||||||||||||

Sbjct 1902 GCGTAAGAGTGCTATCTCCAACTTCCATTA 1931

> [***PifHDAC6*** PITG_01911](http://www.genome.jp/dbget-bin/www_bget?pif:PITG_01911)

Query 30 GCAAAGAGTGAGATGTATTACGCTGTCAACGTCCCACTACACAGCGGCATGGATGAC 89

|||||||||||||||||||||||||||||||||||||||||||||||||||||||||

Sbjct 529 GCAAAGAGTGGGATGTATTACGCTGTCAACTTCCCACTACACAGCGGCATGGATGAC 585

Query 90 GATAGCTACGAAAGCATATTCAAGCCAGTGATAGA 124

|||||||||||||||||||||||||||||||||||

Sbjct 586 GATAGCTACGAAAGCATATTCAAGCCAGTGATAGA 620

> [***PifHDAC7*** PITG_15415](http://www.genome.jp/dbget-bin/www_bget?pif:PITG_15415) **GenBank accession number: KX492581**

Query 137 TCGCGTTCCTGGTCGCGTAGAGCGACCTGAACGCGTGCAAGTTGTGCTAAAGCGTATTAG 196

||||||||||||||||||||||||||||||||||||||||||||||||||||||||||||

Sbjct 325 TCGCGTTCCTGGTCGCGTAGAGCGACCTGAACGCGTGCAAGTTGTGCTAAAGCGTATTAG 266

Query 197 AGAGAGATTCCCACGACTACCGACGCAGACGACGCTTCCTTCAGCGACAGATGAACAGCT 256

|||||||||||||||||||||||||||||||||||||||||||||||||||||||||||||

Sbjct 265 AGAGAGATTCCCACGACTACCGACGCAGACGACGCTTCCTTCAGCGACAGATGAACAGCT 206

Query 257 TCAACGCGCTCACGGAGAAGTGTACATGGATATGGTGGCCGGAATGAGCAATAAAGACTA 316

|||||||||||||||||||||||||||||||||||||||||||||||||||||||||||||

Sbjct 205 TCAACGCGCTCACGGAGAAGTGTACATGGATATGGTGGCCGGAATGAGCAATAAAGACTA 146

Query 317 TGTGGCTAGTCCGAGAGCGAGTAGAATCCCAACAGTTCAGGGAAAG 362

||||||||||||||||||||||||||||||||||||||||||||||

Sbjct 145 TGTGGCTAGTCCGAGAGCGAGTAGAATCCCAACAGTTCAGGGAAAG 100

> [***PifHDAC8*** PITG_21309](http://www.genome.jp/dbget-bin/www_bget?pif:PITG_21309)

Query 30 GCAAAGAGAGAGAAGTATTACGCTGTCAACGTCCCACTACACAGCGGCATGGATGAC 89

|||||||||||||||||||||||||||||||||||||||||||||||||||||||||

Sbjct 496 GCAAAGAGTGGGAAGTATTACGCTGTCAACTTCCCACTACACAGCGGCATGGATGAC 552

Query 90 GATAGCTACGAAAGCATATTCAAGCCAGTGATAGA 124

|||||||||||||||||||||||||||||||||||

Sbjct 553 GATAGCTACGAAAGCATATTCAAGCCAGTGATAGA 587

> [***PifSir2.1*** PITG_10164](http://www.genome.jp/dbget-bin/www_bget?pif:PITG_10164)

Query 14 CGGCGGAGGAGGAAGGAGCTATAGATGCTGATGCCGTAGCAGTAGCGATGGATGCAGACG 73

||||||||||||||||||||||||||||||||||||||||||||||||||||||||||||

Sbjct 1547 CGGCGGAGGAGGAAGGAGCTATAGATGCTGATGCCGTAGCAGTAGCGATGGATGCAGACG 1606

Query 74 ACGACAAGAAAGCGCCTTCGATCGACGAGTATTCAAAGGACGACCAAGACGAACTGATGG 133

||||||||||||||||||||||||||||||||||||||||||||||||||||||||||||

Sbjct 1607 ACGACAAGAAAGCGCCTTCGATCGACGAGTATTCAAAGGACGACCAAGACGAACTGATGG 1666

Query 134 GAAGTGGCAACGAGTTGCTAGGGGCCTCAAGCTT 167

||||||||||||||||||||||||||||||||||

Sbjct 1667 GAAGTGGCAACGAGTTGCTAGGGGCCTCAAGCTT 1700

> [***PifSir2.2*** PITG_06363](http://www.genome.jp/dbget-bin/www_bget?pif:PITG_06363)

Query 6 CAGTGCAAAAATAAACTAGCAGCTGGTATGAATGACGACGATGACGACGAAGAAGAAGAG 62

||||||||||||||||||||||||||||||||||||||||||||||||||||||||||||

Sbjct 1270 CAGTGCAAAAATAAACTAGCAGCTGGTATGAATGACGACGATGACGACGAAGAAGAAGAG 1329

Query 63 AAGCAGCAGACGGAGGAAGAAATTGAGGAGGATATCGTGTGCAATCGTTGCGAACGCCAG 122

||||||||||||||||||||||||||||||||||||||||||||||||||||||||||||

Sbjct 1330 AAGCAGCAGACGGAGGAAGAAATTGAGGAGGATATCGTGTGCAATCGTTGCGAACGCCAG 1389

Query 123 ATTGACACAGAAGCAGAGCC 141

||||||||||||||||||||

Sbjct 1390 ATTGACACAGAAGCAGAGCC 1409

> [***PifSir2.3*** PITG_00718](http://www.genome.jp/dbget-bin/www_bget?pif:PITG_00718)

Query 94 ATTTTAACAAATTGGTGCCCTCCACAACGCACATGGCGCTGTATGAGCTGCATCGACTGG 153

||||||||||||||||||||||||||||||||||||||||||||||||||||||||||||

Sbjct 407 ATTTTAACAAATTGGTGCCCTCCACAACGCACATGGCGCTGTATGAGCTGCATCGACTGG 466

Query 154 GCTACTTGAAGCACGTCGTGTCCCAAAATGTGGACAATCTGCATCTAAAAAGTGGCGT 211

||||||||||||||||||||||||||||||||||||||||||||||||||||||||||

Sbjct 467 GCTACTTGAAGCACGTCGTGTCCCAAAATGTGGACAATCTGCATCTAAAAAGTGGCGT 524

> [***PifEF1*** PITG_06722](http://www.genome.jp/dbget-bin/www_bget?pif:PITG_06722) **GenBank accession number: KX492582**

Query 7 CCCGACTTGACGAACTTGGGGGCAGTCTCGAGCACCTTGCCCGAACGACGGTCCATCTTC 65

||||||||||||||||||||||||||||||||||||||||||||||||||||||||||||

Sbjct 1154 CCCGACTTGACGAACTTGGGGGCAGTCTCGAGCACCTTGCCCGAACGACGGTCCATCTTC 1095

Query 66 TCCGTAATCTCTTTAAACTTGCAGGCAACGTGGGCCGTGTGGCAGTCAAGCACAGGCGAG 125

||||||||||||||||||||||||||||||||||||||||||||||||||||||||||||

Sbjct 1094 TCCGTAATCTCTTTAAACTTGCAGGCAACGTGGGCCGTGTGGCAGTCAAGCACAGGCGAG 1035

Query 126 TAACCGTTGCCGATCTGACCAGGGTGGTTCAGCACAATCACCTGGGCGGTGAAGTCCTGG 185

||||||||||||||||||||||||||||||||||||||||||||||||||||||||||||

Sbjct 1034 TAACCGTTGCCGATCTGACCAGGGTGGTTCAGCACAATCACCTGGGCGGTGAAGTCCTGG 975

Query 186 GTTGCCTTAGCAGGGTCGTTCTTGGA 211

||||||||||||||||||||||||||

Sbjct 974 GTTGCCTTAGCAGGGTCGTTCTTGGA 949
