# Supplementary figures and images for: Diversity, evolution and expression profiles of histone acetyltransferases and deacetylases in oomycetes
Source: BMC Genomics. 2016 Nov 16;17:927. doi: 10.1186/s12864-016-3285-y (PMC5112689; doi:10.1186/s12864-016-3285-y)

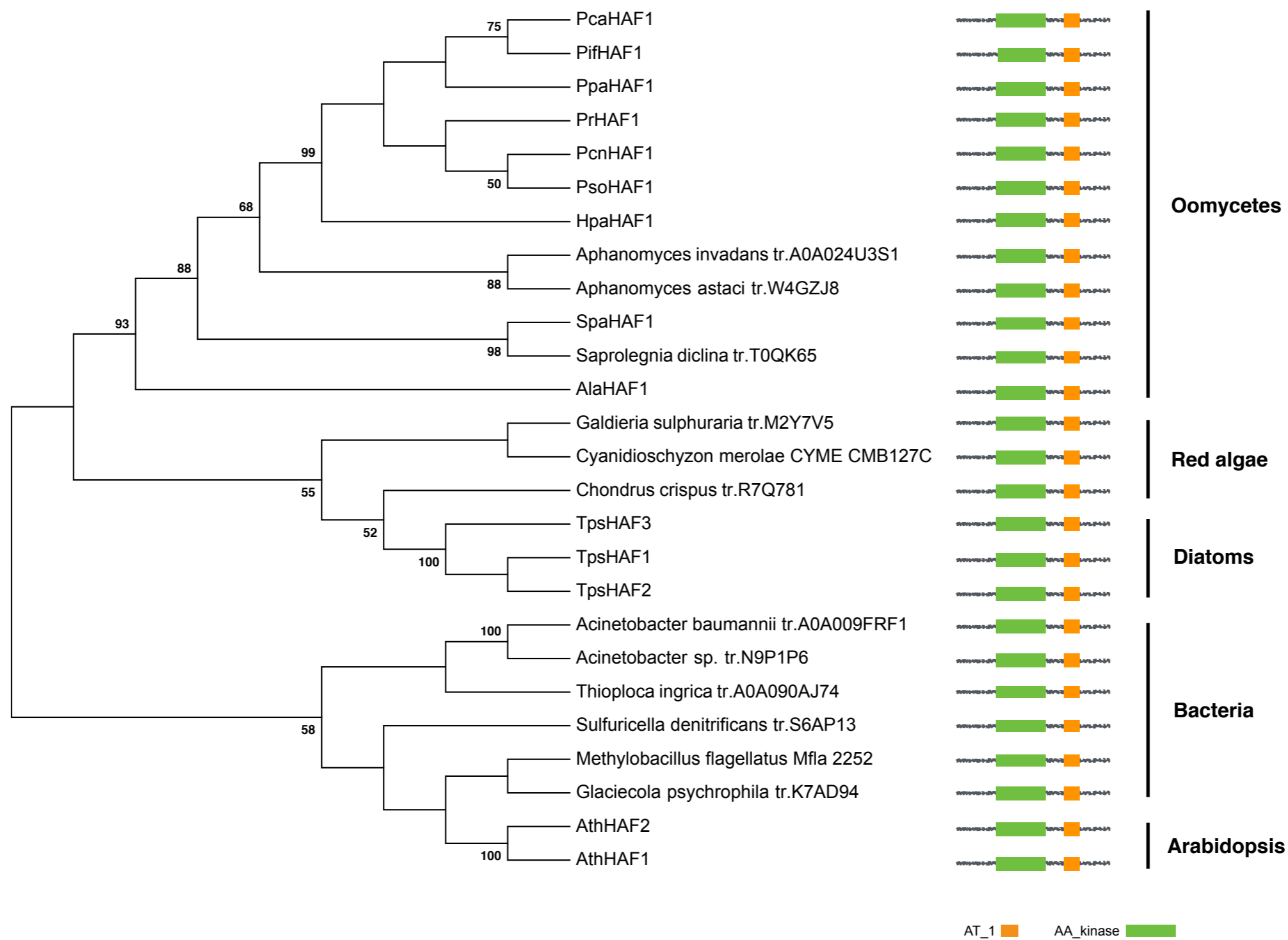

Supplement: Additional file 6: — Phylogenetic tree of HAFs in oomycetes. A maximum-likelihood phylogenetic tree was constructed with sequences of HAF conserved domains from the species described in Fig. 2. Each domain was highlighted by one color and bootstrap values (≥50%) are shown near the tree nodes. (PDF 194 kb) [file 12864_2016_3285_MOESM6_ESM.pdf]

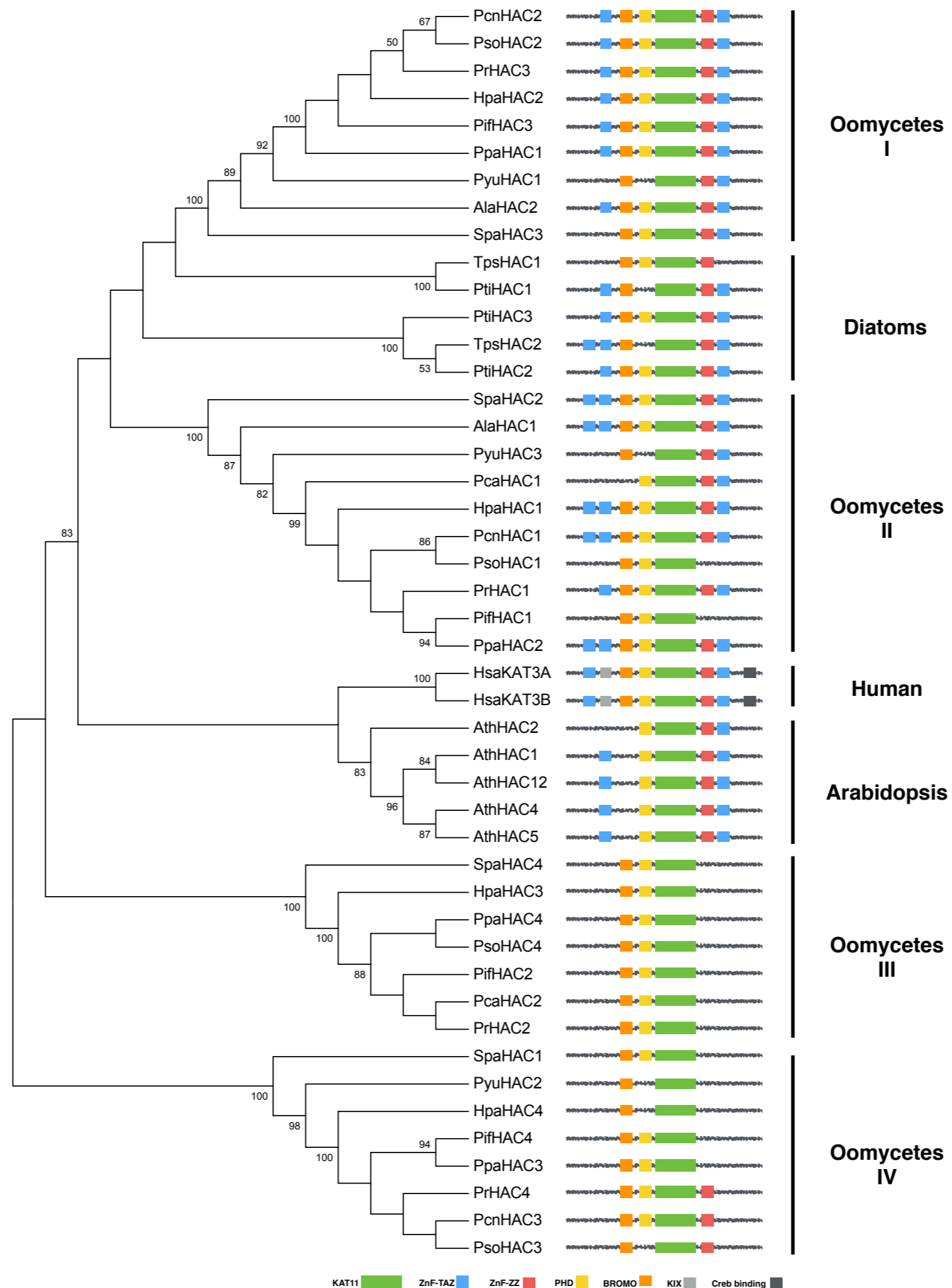

Supplement: Additional file 7: — Phylogenetic tree of HACs in oomycetes. A maximum-likelihood phylogenetic tree was constructed with sequences of HAC conserved domains from the species described in Fig. 2. All the predicted HACs of oomycetes were attributed to four clades according to the combination of conserved domains. These domains were highlighted by different colors and bootstrap values (≥50%) are shown near the tree nodes. (PDF 423 kb) [file 12864_2016_3285_MOESM7_ESM.pdf]

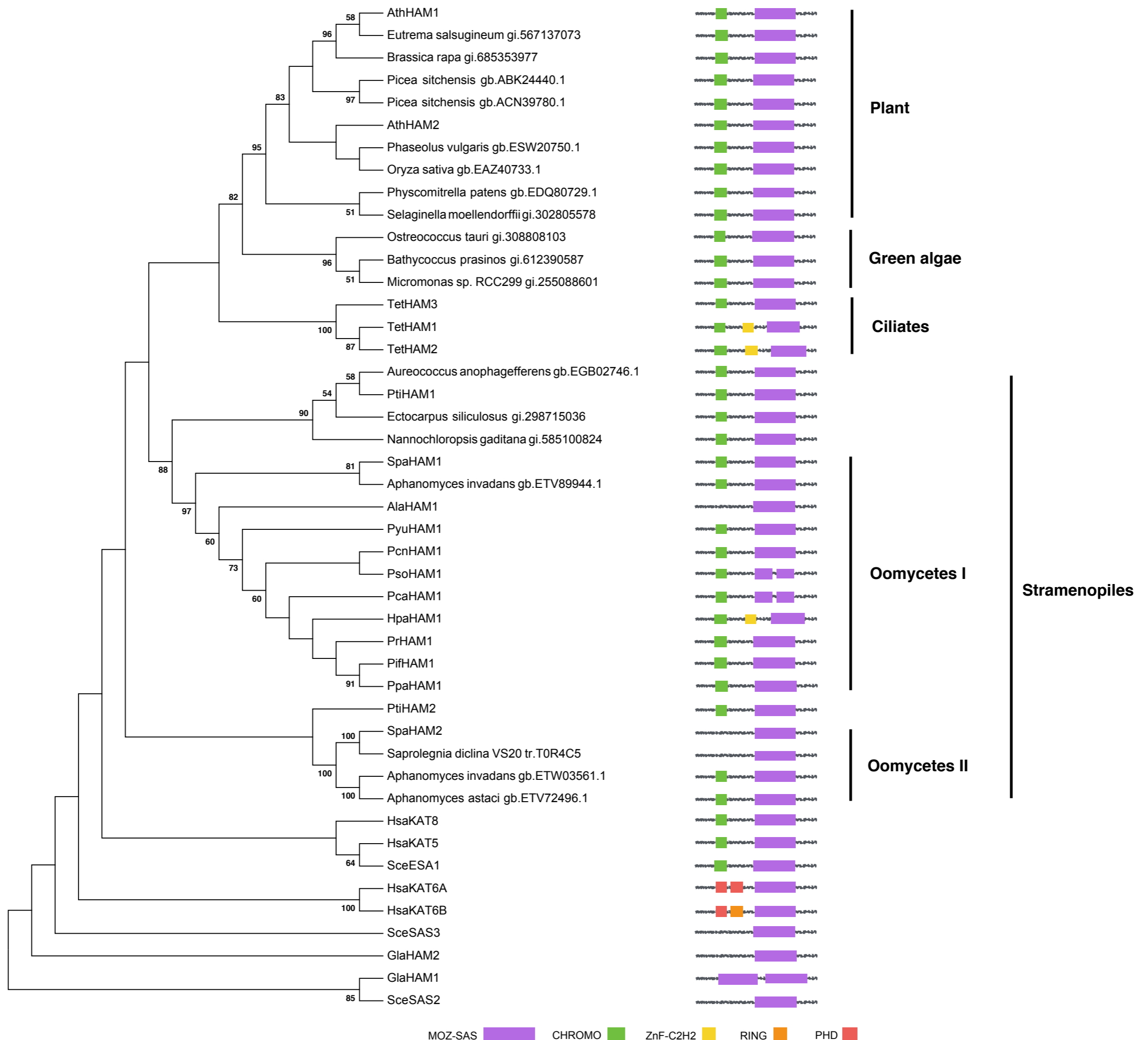

Supplement: Additional file 8: — Phylogenetic tree of HAMs in oomycetes. A maximum-likelihood phylogenetic tree was constructed with sequences of HAM conserved domains from the species described in Fig. 2. Different domains were highlighted by different colors and bootstrap values (≥50%) are shown near the tree nodes. (PDF 303 kb) [file 12864_2016_3285_MOESM8_ESM.pdf]

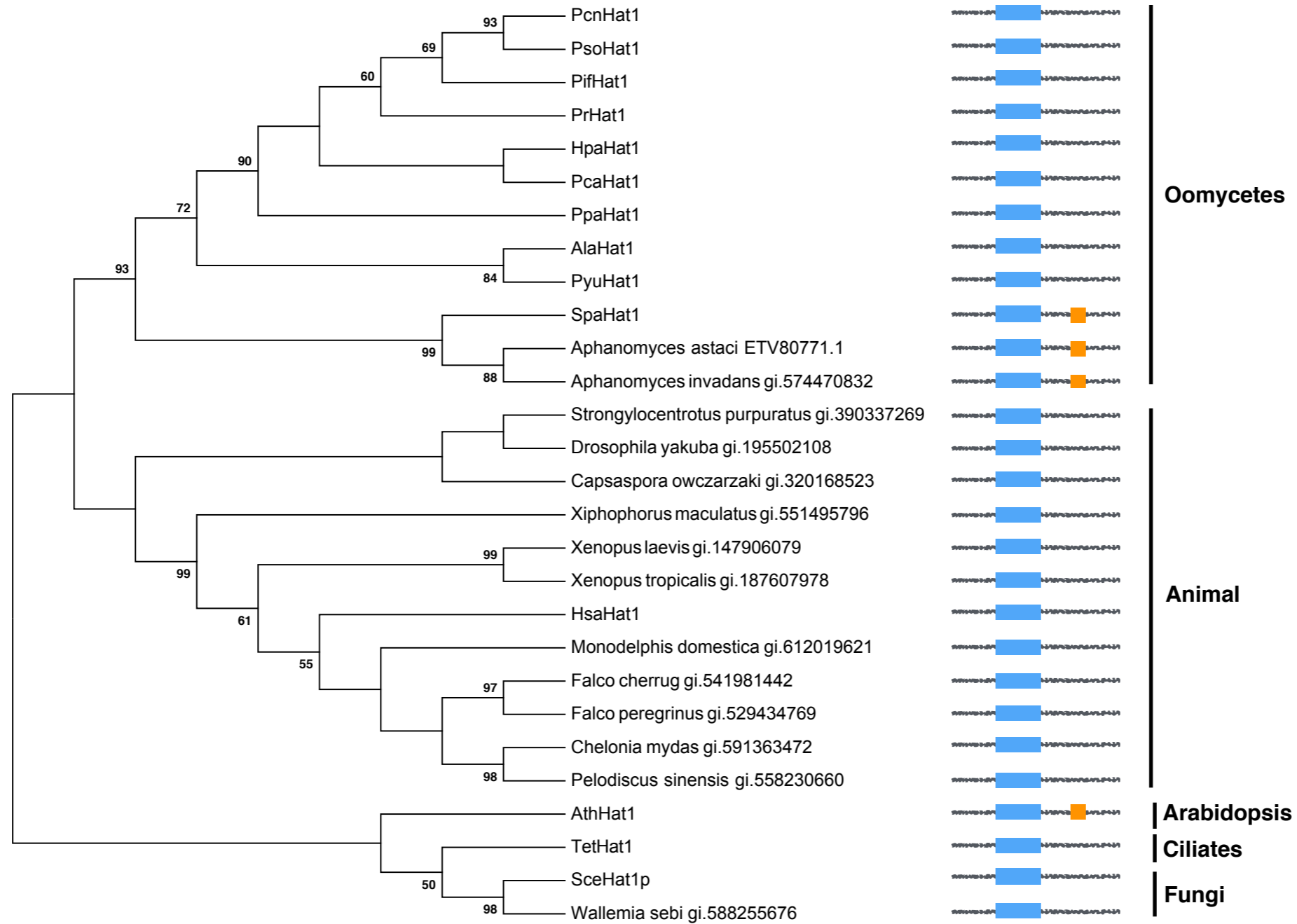

Hat1\_N ■ AT\_1 ■

Supplement: Additional file 9: — Phylogenetic tree of Hat1s in oomycetes. A maximum-likelihood phylogenetic tree was constructed with sequences of Hat1 conserved domains from the species described in Fig. 2. Each Oomycetes species contained one Hat1 and they were attributed to 1 clade. Each domain was highlighted by one color and bootstrap values (≥50%) are shown near the tree nodes. (PDF 201 kb) [file 12864_2016_3285_MOESM9_ESM.pdf]

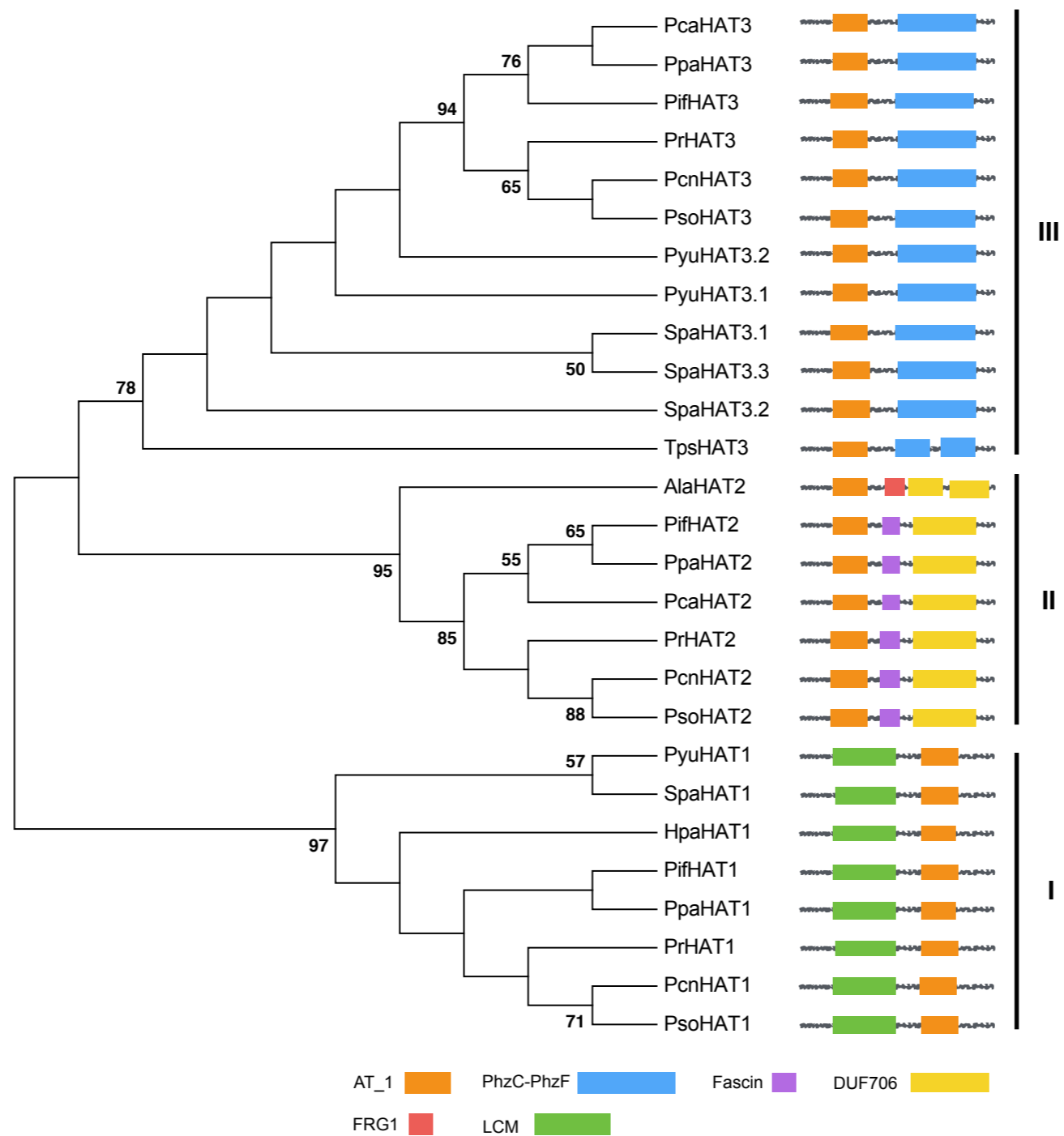

Supplement: Additional file 10: — Phylogenetic tree of putative novel HATs in oomycetes. A maximum-likelihood phylogenetic tree was constructed with sequences of the conserved domain AT-1 found in the species described in Fig. 2. Different domains were highlighted by different colors and bootstrap values (≥50%) are shown near the tree nodes. (PDF 189 kb) [file 12864_2016_3285_MOESM10_ESM.pdf]

Pea agar  
V8 agar  
RS agar  
2dpi  
3dpi  
4dpi  
5dpi

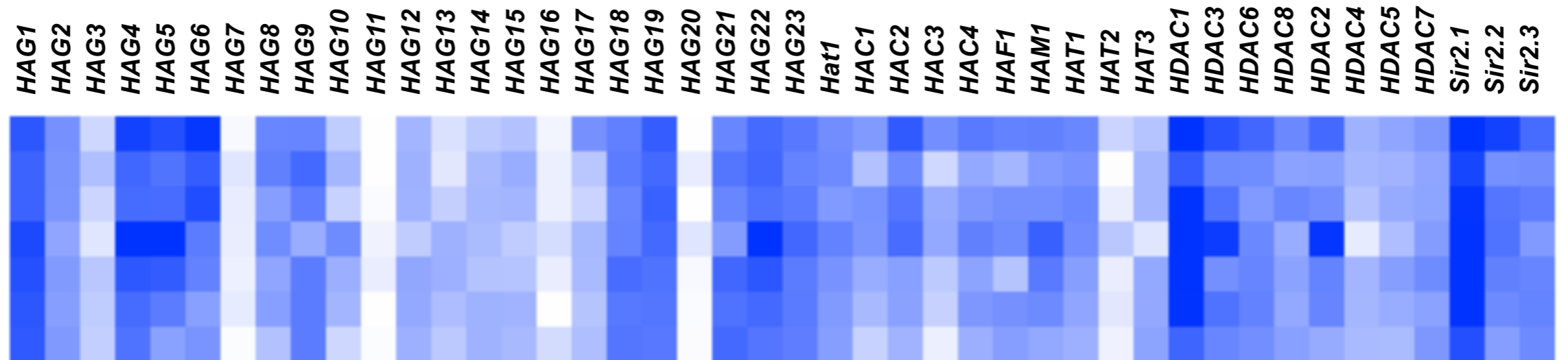

9.0

12.0

Supplement: Additional file 13: — Expression profiles of HATs and HDACs in P. infestans at various stages during infection. The heat map generated by microarray expression values of GSE14480 retrieved from GEO DataSets. The color bar represents the log2 of expression values, and the highest and lowest log2 signal value is 8.62 and 12.97, respectively. (PDF 62 kb) [file 12864_2016_3285_MOESM13_ESM.pdf]
