# Supplementary material for: Diversity, evolution and expression profiles of histone acetyltransferases and deacetylases in oomycetes
Source: BMC Genomics. 2016 Nov 16;17:927. doi: 10.1186/s12864-016-3285-y (PMC5112689; doi:10.1186/s12864-016-3285-y)
Supplement: Additional file 11: — Sequence alignment of some Hpa2s in oomycetes. Bootstrap values (≥30%) and sequence identity are shown near the tree nodes. a Members of Hpa2 in the same species with high sequence identity aggregated in the same scaffold. b Members of Hpa2 in the same species with various sequence identities clustered in the same scaffold. (PDF 421 kb) [file 12864_2016_3285_MOESM11_ESM.pdf]

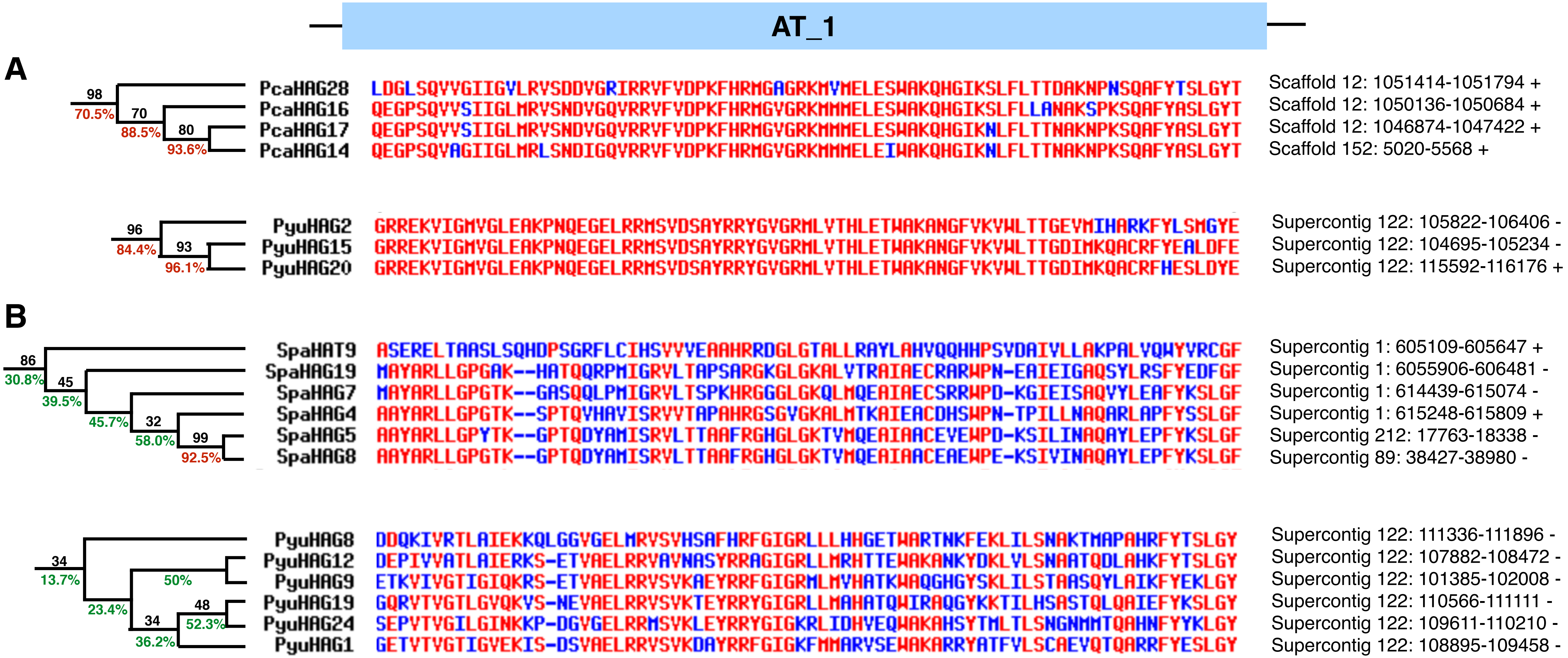

AT_1
A
PcaHAG28 LDGLSQVVGIIGVLRVSDDVGRIRRVFVDPKFHRMGAGRKMVMELESWAKQHGIKSLFLTTDAKNPNSQAFYTSLGYT Scaffold 12: 1051414-1051794 +
PcaHAG16 QEGPSQVVSIIGLMRVSNDVGQVRRVFVDPKFHRMGVGRKMMMELESWAKQHGIKSLFLLANAKSPKSQAFYASLGYT Scaffold 12: 1050136-1050684 +
PcaHAG17 QEGPSQVVSIIGLMRVSNDVGQVRRVFVDPKFHRMGVGRKMMMELESWAKQHGIKNLFLTTNAKNPKSQAFYASLGYT Scaffold 12: 1046874-1047422 +
PcaHAG14 QEGPSQVAGIIGLMRLSNDIGQVRRVFVDPKFHRMGVGRKMMMELEIWAKQHGIKNLFLTTNAKNPKSQAFYASLGYT Scaffold 152: 5020-5568 +
98
70.5%
70
88.5%
80
93.6%
PyuHAG2 GRREKVIGMVGLEAKPNQEGELRRMSVDSAYRRYGVGRMLVTHLETWAKANGFVKVWLTTGEVMIHARKFYLSMGYE Supercontig 122: 105822-106406 -
PyuHAG15 GRREKVIGMVGLEAKPNQEGELRRMSVDSAYRRYGVGRMLVTHLETWAKANGFVKVWLTTGDIMKQACRFYEALDFE Supercontig 122: 104695-105234 -
PyuHAG20 GRREKVIGMVGLEAKPNQEGELRRMSVDSAYRRYGVGRMLVTHLETWAKANGFVKVWLTTGDIMKQACRFHESLDYE Supercontig 122: 115592-116176 +
96
84.4%
93
96.1%
B
SpaHAT9 ASERELTAASLSQHDPSGRFLCIHSVVVEAAHRRDGLGTALLRAYLAHVQQHHPSVDAIVLLAKPALVQWYVRCGF Supercontig 1: 605109-605647 +
SpaHAG19 MAYARLLGPGAK--HATQQRPMIGRVLTAPSARGKGLGKALVTRAIAECRARWPN-EAIEIGAQSYLRSFYEDFGF Supercontig 1: 6055906-606481 -
SpaHAG7 MAYARLLGPGTK--GASQQLPMIGRVLTSPKHRGGGLGKQLMQEAIAECSRRWPD-KGIEISAQVYLEAFYKSLGF Supercontig 1: 614439-615074 -
SpaHAG4 AAYARLLGPGTK--SPTQVHAVISRVVTAPAHRGSGVGKALMTKAIEACDHSWPN-TPILLNAQARLAPFYSSLGF Supercontig 1: 615248-615809 +
SpaHAG5 AAYARLLGPYTK--GPTQDYAMISRVLTTAAFRGHGLGKTVMQEAIAACEVEWPD-KSILINAQAYLEPFYKSLGF Supercontig 212: 17763-18338 -
SpaHAG8 AAYARLLGPGTK--GPTQDYAMISRVLTTAAFRGHGLGKTVMQEAIAACEAEWPE-KSIVINAQAYLEPFYKSLGF Supercontig 89: 38427-38980 -
86
30.8%
45
39.5%
45.7%
32
58.0%
99
92.5%
PyuHAG8 DDQKIVRTLAIEKKQLGGVGELMRVSVHSAFHRFGIGRLLLHHGETWARTNKFEKLILSNAKTMAPAHRFYTSLGY Supercontig 122: 111336-111896 -
PyuHAG12 DEPIVVATLAIERKS-ETVAELRRVAVNASYRRAGIGRLLMRHTTEWAKANKYDKLVLSNAATQDLAHKFYTSLGY Supercontig 122: 107882-108472 -
PyuHAG9 ETKVIVGTIGIQKRS-ETVAELRRVSVKAEYRRFGIGRMLMVHATKWAQGHGYSKLILSTAASQYLAIKFYEKLGY Supercontig 122: 101385-102008 -
PyuHAG19 GQRVTVGTLGVQKVS-NEVAELRRVSVKTEYRRYGIGRLLMAHATQWIRAQGYKKTILHSASTQLQAIEFYKSLGY Supercontig 122: 110566-111111 -
PyuHAG24 SEPVTVGILGINKKP-DGVGELRRMSVKLEYRRYGIGKRLIDHVEQWAKAHSYTMLTLSNGNMMTQAHNFYYKLGY Supercontig 122: 109611-110210 -
PyuHAG1 GETVTVGTIGVEKIS-DSVAELRRVSVKDAYRRFGIGKFMMARVSEWAKARRYATFVLSCAEVQTQARRFYESLGY Supercontig 122: 108895-109458 -
34
13.7%
50%
23.4%
48
52.3%
34
36.2%
